# Neighbour-induced changes in root exudation patterns of buckwheat results in altered root architecture of redroot pigweed

**DOI:** 10.1038/s41598-024-58687-3

**Published:** 2024-04-15

**Authors:** Çağla Görkem Eroğlu, Alexandra A. Bennett, Teresa Steininger-Mairinger, Stephan Hann, Markus Puschenreiter, Judith Wirth, Aurélie Gfeller

**Affiliations:** 1 Herbology in Field Crops, Plant Production Systems, Agroscope, Nyon, Switzerland; 2 Department of Chemistry, Institute of Analytical Chemistry, University of Natural Resources and Life Sciences, Vienna (BOKU), 1190 Vienna, Austria; 3 Department of Forest and Soil Sciences, Institute of Soil Research, Rhizosphere Ecology & Biogeochemistry Group, University of Natural Resources and Life Sciences, Vienna, Konrad-Lorenz-Strasse 24, 3430 Tulln, Austria

**Keywords:** Split-root system, Root exudate, Plant interaction, Metabolomics, Weed, Root system architecture, Ecology, Plant sciences

## Abstract

Roots are crucial in plant adaptation through the exudation of various compounds which are influenced and modified by environmental factors. Buckwheat root exudate and root system response to neighbouring plants (buckwheat or redroot pigweed) and how these exudates affect redroot pigweed was investigated. Characterising root exudates in plant–plant interactions presents challenges, therefore a split-root system which enabled the application of differential treatments to parts of a single root system and non-destructive sampling was developed. Non-targeted metabolome profiling revealed that neighbour presence and identity induces systemic changes. Buckwheat and redroot pigweed neighbour presence upregulated 64 and 46 metabolites, respectively, with an overlap of only 7 metabolites. Root morphology analysis showed that, while the presence of redroot pigweed decreased the number of root tips in buckwheat, buckwheat decreased total root length and volume, surface area, number of root tips, and forks of redroot pigweed. Treatment with exudates (from the roots of buckwheat and redroot pigweed closely interacting) on redroot pigweed decreased the total root length and number of forks of redroot pigweed seedlings when compared to controls. These findings provide understanding of how plants modify their root exudate composition in the presence of neighbours and how this impacts each other’s root systems.

## Introduction

Plant roots adapt and cope with alterations in environmental conditions; their growth and spatial distribution patterns change in the presence of other plants. The focus of previous research on belowground plant–plant interactions and neighbour detection was mostly on those that occur passively through alterations in the environment and fluctuations in the availability of resources, such as light, nutrients and water, which are driven by the presence of neighbouring plants and competition^1–3^. These physical signals and responses are widely studied; It is known that plants have the capability of utilising resources and depleting the shared environment, consequently affecting the growth, development, and survival of their neighbours. This physical competition, in addition to rhizospheric chemical communication, allows certain plants known as cover crops to naturally suppress weeds in agricultural settings^4–6^.

However, a debate has developed in the literature about this rhizospheric communication on whether plants have belowground self and non-self-recognition and has attempted to conceptualize this interaction with several terms and definitions^7,8^. Root–root interactions can occur either interspecifically, between the members of different plant species, or intraspecifically, within the individuals of the same species^3,9,10^. As the changes in root morphology may vary depending on environmental conditions, it is difficult to conclude the general response(s) caused specifically by their neighbours^11,12^. Though it is difficult to parse what causes these responses, there is increasing evidence that plants actively generate and use chemical signalling by producing root exudates and volatile organic compounds which affect other organisms, including plant neighbours, in their vicinity^13–16^.

These root exudates can be classified as primary metabolites (e.g., amino acids, sugars, and organic acids) directly involved in growth and development, and specialized metabolites (e.g., phenolics, terpenoids) involved in functions such as defence and attraction. Alternatively, they can be distinguished in three groups according to their molecular weight as low molecular weight compounds (amino acids, organic acids, sugars, phenolics, and a wide variety of secondary compounds), high molecular weight compounds (polysaccharides and proteins), and ions. Exudates can be released via different mechanisms such as secretion, diffusion (low molecular weight compounds), and excretion (e.g., mucilage)^17–21^. The concentration and composition of root exudates depend on many factors such as the plant species and abiotic and biotic factors including the microbial and plant community present^22,23^. After release into the soil environment, root exudates may (i) directly influence the metabolism of neighbour species, (ii) be consumed, degraded, or transformed by plants or soil microorganisms and/or (iii) change the physical and chemical soil properties^20,24–26^. There are some well-studied compounds known to have inhibiting effects on germination and growth of other plants species^27^, such as momilactones in rice^28,29^, benzoxazinoids in cereals such as rye^25,30^, wheat^31^, and maize^32^, hordenine and gramine in barley^33^ and sorgoleone in sorghum^34^.

While some compounds in root exudates have been isolated and well identified, specifically characterising altered composition of root exudates in response to a plant neighbour has shown to be difficult when multiple plant species are grown together. Split-root systems require the division of one plant root system into multiple compartments. This enables the application of differential treatments to each compartment (e.g., different plant neighbours can be present or absent in each compartment). There are different ways to set up a split-root system depending on the factors such as the aim of the study, plant age required for the treatment, and/or the type plant and its root system^35,36^. The availability of such diverse techniques to establish split-root systems in different plant species while treating evenly divided parts of a single root system differentially is useful for various research topics (e.g., plant nutrient uptake and transport^37–39^, abiotic and biotic stress^40–43^, hormone signalling^44–46^, and symbioses with soil microbes^47^). Additionally, split-root systems serve as a useful tool in plant root–root interaction studies. It allows half of the root system to be exposed to the roots of a neighbour while the other half is not. This system enables the isolation and assessment of exudates from a single plant species when it is grown in the presence of another. A thorough search of the relevant literature indicates that this is the first study on split-root interactions of buckwheat at the time of publication.

Buckwheat (BK) (*Fagopyrum esculentum* Moench) belongs to the Polygonaceae family and forms a taproot with a dense root system^48^. Cover crops such as BK are used in agriculture for their multiple ecosystem services such as reducing soil erosion, improving soil properties, attracting pollinators/beneficial insects, and weed suppression^49–51^. Weeds cause substantial yield losses^52^ and using herbicides for weed management is neither sustainable nor environmentally friendly due to evolved herbicide resistance and toxicity. Therefore, it is pertinent to decrease the use of herbicides and exploit all levels of integrated weed management^53^. Cover crops can suppress weeds indirectly through competition for resources and directly by releasing compounds. In previous studies, BK showed suppressive effects on the growth of redroot pigweed (P) (*Amaranthus retroflexus* L.), a troublesome dicot weed species native to North America and currently distributed nearly worldwide. The observed growth suppression was due to both shading and root–root interactions^27,54,55^. However, in field trials, the shading effect of BK was not the principal growth suppressive factor for P^56^. Studies performed targeted metabolomic analysis have shown that BK produces numerous flavonoids and phenolics, such as (+)-catechin, 4-hydroxyacetophenone, vanillic acid, and gallic acid, which exhibit inhibitory effects on various weed species^57–59^.

While many studies perform targeted metabolomics to identify known compounds to a high level of certainty, a non-targeted analysis (NTA) approach has the advantage of recovering as many relevant chemical signals as possible from a sample. This allows for the discovery of novel, and often unknown, compounds not previously considered and the description of complex changes in exudate patterns of many compounds^60^. However, it is not possible to generate precise absolute quantification and fragmentation pattern information for all these compounds during the NTA^61^. It is for this reason that NTA is considered an exploratory tool that benefits from follow up targeted analysis for validation^62^.

**Figure 1:**
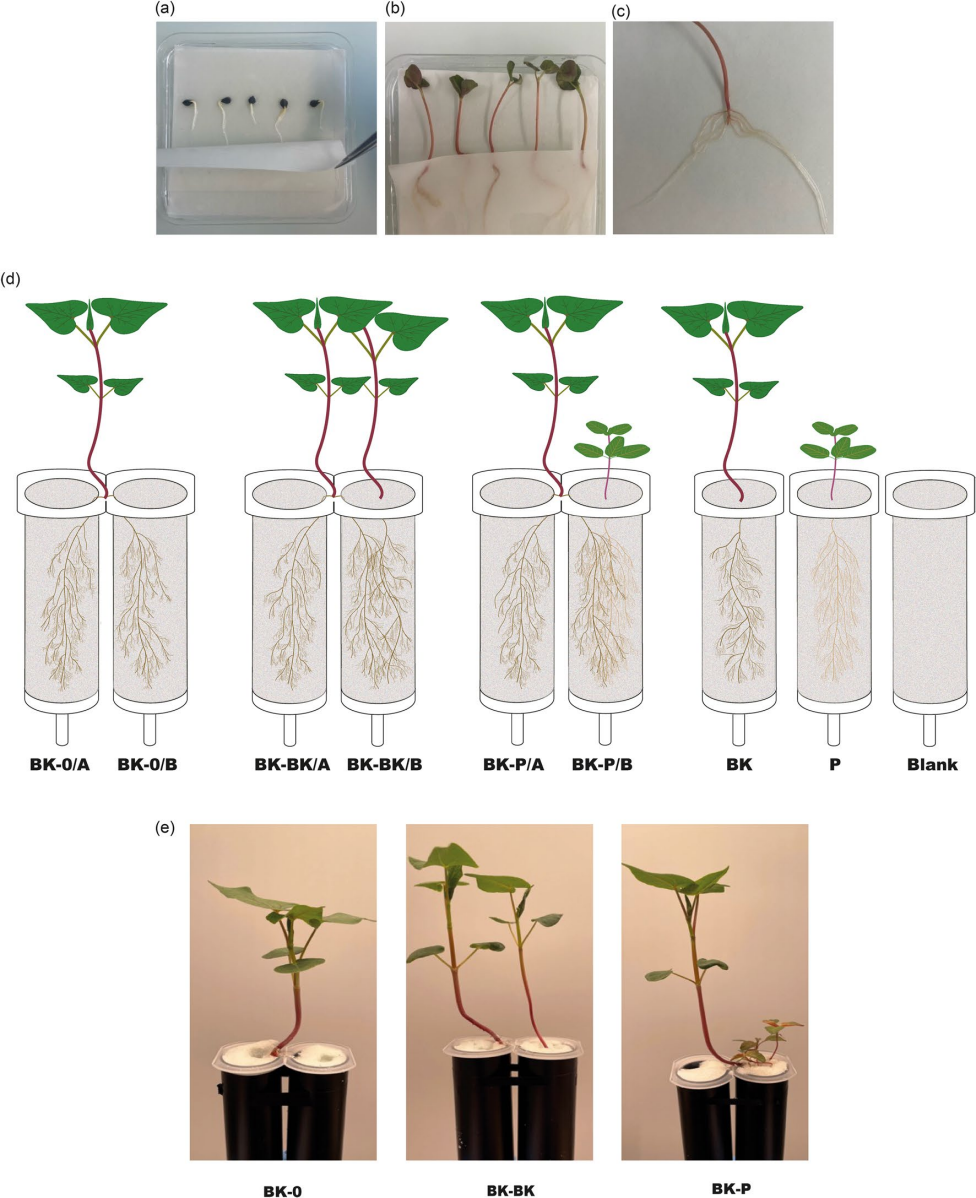
Experimental setup of the buckwheat (BK) split-root system. Germinated BK seeds on filter paper wetted with half strength Hoagland solution in Petri dish on day three, just before cutting the roots (**a**), 7-day old BK seedlings, just before the transfer to split-root systems (**b**), BK roots split into two equal parts to be able to transfer them into split-root system (**c**) representation of each growth condition (**d**): **BK-0**: BK split-root without any neighbour, half of the root system is in compartment A (BK-0/A) and the other half is in compartment B (BK-0/B). **BK-BK**: BK split-root with a homospecific BK neighbour, half of the root system is in compartment A without root contact to the BK neighbour (BK-BK/A), and the other half is in compartment B with root contact to the BK neighbour (BK-BK/B). **BK-P: BK** split-root with a heterospecific redroot pigweed (P) neighbour, half of the root system is in compartment A without root contact to the P neighbour (BK-P/A) and the other half is in compartment B with root contact to the P neighbour (BK-P/B). **BK** and **P**: non split BK and P. **Blank**: cartridge filled with glass beads and no plants (**d**), BK split-root systems on 14th day after the transfer (**e**).

With the interest of using an NTA approach to explore belowground plant-plant communication dynamics, our objectives were: (i) to investigate whether BK perceives the presence of intra- (BK) and interspecific (P) neighbours through interacting root systems and systematically modify its root exudate composition, as well as root system architecture; (ii) to determine if these changes in root exudates unique to the species tested and/or are a general response to the presence of either neighbouring plant; and (iii) to assess the effects of root exudates obtained from BK interacting with neighbours on P root system architecture. To fulfill these objectives, we developed a BK split-root system (Fig. 1) and a biological test enabling root exudate collection and root exudate application on P, respectively. In a non-targeted screening approach, exudates were chemically analysed using ultra-high performance liquid chromatography (UHPLC) paired with high-resolution mass spectrometry (HRMS) and statistically analysed to assess differentially expressed compounds.

## Results

### Non-targeted metabolomics profiling of root exudates

#### Normalisation

Dry root weight is the most frequently employed parameter for normalisation in root metabolism studies^63,64^. However, there is no evidence in the literature that a higher dry root biomass is necessarily related to higher root exudation. Therefore, we compared dry root weight and the number of root tips and postulated that if the root exudation primarily takes place at the root tips^11^ then the number of root tips might be a better parameter for biological normalisation.

**Figure 2:**
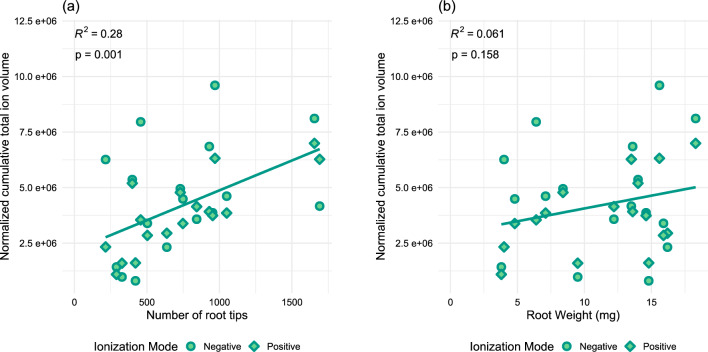
Linear regressions of different data parameters showing correlation between number of root tips and normalised cumulative total compound signal (**a**) and dry root weight in mg and normalised cumulative total compound signal (**b**). Data was restricted to samples of non-split root BK (BK) and split root BK isolated compartment (A) of BK grown with no neighbour (BK-0), BK with a same species neighbour (BK-BK), and BK with a P neighbour (BK-P) (*n* = 3–5/condition). Data was normalised first through use of internal standard (3,5-Di-tert-butyl-4-hydroxybenzoic acid) and pooled quality control samples with the LOWESS method in MS DIAL. Total compound signal (TCS) was calculated by annotating adducts and then combining adduct and non-adduct signals of the same compound. All signals were added together within a single sample to create a cumulative value to represent total metabolite signal. Positive and negative ionisation mode data is combined in these figures.

The two biological parameters were correlated with cumulative total compound signal (TCS). The correlation between the number of root tips and cumulative TCS (Fig. 2a, R^2^ = 0.28, p = 0.001) was stronger than the correlation between root weight and cumulative TCS (Fig. 2b, R^2^ = 0.061, p = 0.158). Therefore, instead of root weight, the number of root tips was used for normalisation.

#### Dimension reduction and multivariate statistical analysis of metabolites

**Figure 3:**
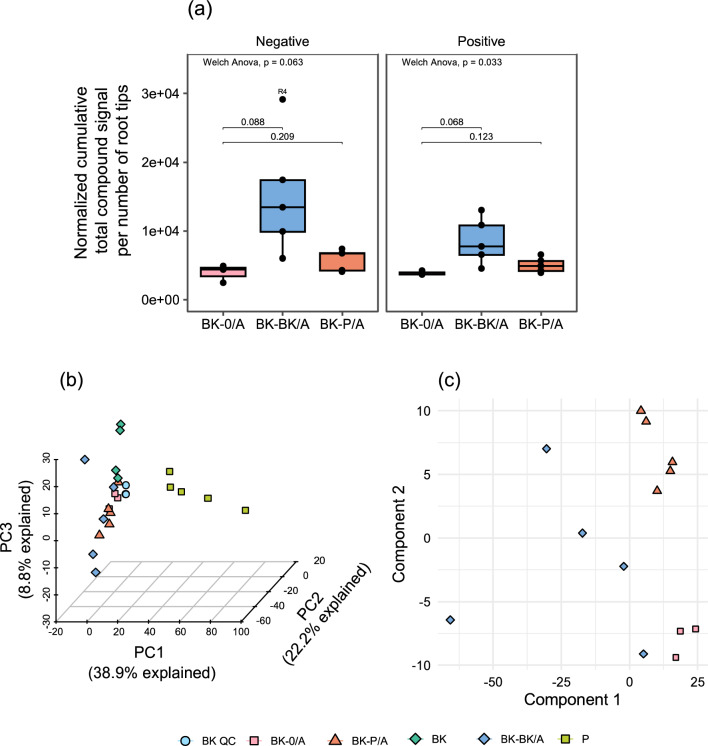
Data dimension reduction to observe holistic changes in metabolite expression. Data comes from BK with split roots grown in two compartments with (compartment B) and without (compartment A) contact to a neighbour plant. The three conditions with no neighbour (BK-0/A), with a same species neighbour (BK-BK/A), and with a P neighbour (BK-P/A) are shown. Data from non-split root samples of BK and P are included in multivariate analysis. *n* = 3–5/condition. Quality control (QC) samples are a mixture of all sample conditions. Data from the shared compartment of the BK split-root setup where P is the neighbour (BK-P/B) is not included in these analyses due to inability to normalise data biologically. Variables consist of total compound signal (TCS) features normalised biologically by the number of root tips. When comparing BK-0/A to BK-BK/A or BK-P/A via box and whisker plot, TCS features are summed into a cumulative value to reduce the dimensionality of the data to one variable and represent total metabolite signal. This shows there was a significant difference among groups using Welch's ANOVA. But significance was lost after performing a Dunnett’s T3 post hoc test modified to use BK-0/A as a control group (**a**). A three-dimensional PCA was performed utilising all detected metabolites exudated (**b**). A PLS-DA highlights differences between the three split-root conditions in which the cover crop roots have been isolated in compartment A (**c**). Both negative and positive ionisation mode data were used to generate multivariate models. Data is centred and auto scaled before performing PCA and PLS-DA.

The within group data distribution of root exudate cumulative TCS from A compartments of the three split-root conditions that contain only half of the BK root systems with no neighbour (BK-0/A), a homospecific neighbour (BK-BK/A), and a heterospecific redroot pigweed neighbour (BK-P/A) was compared (Fig. 3a) to see if there were large changes in total root exudation. Variation in the data was explained by the treatments when analysed with a Welch's ANOVA (p-value = 0.033) in positive ionisation mode, but significance was lost after performing a Dunnett’s T3 post hoc test modified to use BK-0/A as a control group. At this point, no definitive differences among these groups could be observed.

Multivariate analysis was performed to assess the perception of intra- (BK) and interspecific (P) neighbours by BK. A principal component analysis (PCA) of the TCS signals utilising 3 components highlights several key results (Fig. 3b). The three-dimensional PCA was able to account for ~ 70% of variance in the data. QC samples clustered together; this validated the data acquisition and processing pipelines. P samples separated out clearly from BK samples. BK-BK/A overlapped with all other BK conditions. Lastly, BK and BK-0/A samples clustered next to each other, indicating that the samples were similar to one another and that the split-root system alone did not induce major changes in metabolite profile. Since both cumulative TCS box and whisker plots and PCA analysis was not able to visualize much difference between the A compartments of the three split-root conditions, a supervised dimension reduction method, partial least square discriminant analysis (PLS-DA), was utilised to visualize possible difference in root exudation. The three groups did separate out with the PLS-DA, indicating that there were nuance differences between the groups (Fig. 3c).

#### Univariate assessment of individual metabolites

**Figure 4:**
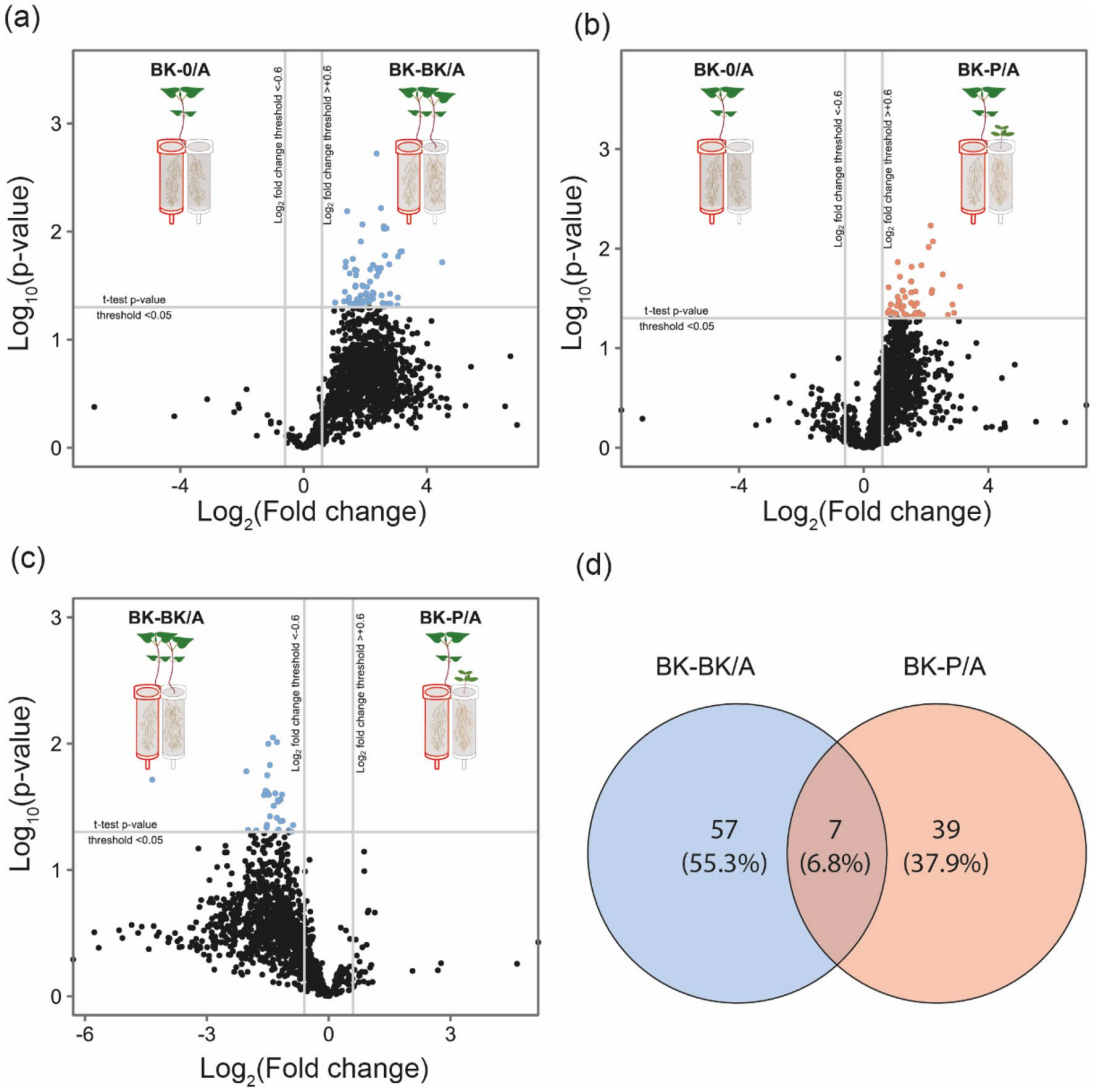
Volcano plots comparing differential expression of metabolites, represented by total compound signal compounds detected by LC-HRMS/MS. Each point corresponds to one detected metabolite. Significance (p < 0.05, *n* = 3–5/condition) was calculated using a Welch’s t-test. The -log_10_ of the p-values was calculated to better visualise the volcano plot. A log_2_ fold change greater than 0.6 or less than -0.6 was used as a significance threshold. Negative and positive ionisation mode data is merged in these figures. Differential expression when comparing compartment, A of BK split-root systems with no neighbour (BK-0/A) to a homospecific BK neighbour (BK-BK/A) (**a**) or a heterospecific P neighbour (BK-P/A) (**b**) shows there are no metabolites which are significantly more expressed under BK-0/A conditions compared to BK-BK/A or BK-P/A conditions. A Venn diagram was utilised to show the number of metabolites upregulated by a BK-P/A and/or BK-BK/A when each is compared to BK-0/A (**d**).

To assess which metabolites were specifically different between groups, univariate statistics was performed on 597 compounds in negative ionization mode and 689 in positive ionization mode. When comparing A compartments of the three split-root conditions, there were no metabolites which were exuded more by buckwheat grown with no neighbour (BK-0/A) in comparison to a buckwheat neighbour (BK-BK/A) and/or a redroot pigweed neighbour (BK-P/A). However, the presence of any neighbour did increase or induce the production of certain metabolites. When compared to BK-0/A, 64 metabolites were upregulated in BK-BK/A (Fig. 4a and Table S1a) and 46 metabolites were upregulated in BK-P/A (Fig. 4b and Table S1b). The presence of a buckwheat neighbour (BK-BK/A) resulted in more compounds being upregulated when compared to a redroot pigweed neighbour (BK-P-A). This is indicated by the significantly elevated expression of 29 metabolites in BK-BK/A when compared to BK-P/A (Fig. 4c and Table S1c).

A Venn diagram was used to show the number of compounds upregulated uniquely by the presence of each neighbour in comparison to BK-0/A (Fig. 4d) to address if upregulation is species-specific recognition and/or a general response. While there was some overlap (~ 7% of the 103 metabolites upregulated from BK-0/A), ~ 55% of the upregulated metabolites were unique to BK-BK/A and ~ 38% were unique to BK-P/A.

Species specific compounds were also of interest. For example, unknown BK metabolite 813 (ionisation mode = positive, m/z = 327.1049, RT = 10.45) in Fig. S2a, while not significantly different for all BK conditions compared to P, is significantly different between non-split BK grown alone and P grown alone. However, metabolites exclusive to P were usually expressed in much higher abundance and easier to statistically annotate. For example, the unknown metabolite 341 (ionization mode = positive, m/z = 203.0810, RT = 4.02) shown in Fig. S2b is significantly (Games-Howell p < 0.05) higher in both BK-P/B and P conditions when compared to all other conditions.

Compounds identified to confidence level 1 according to the Schymanski scale^65^ utilizing the mock root exudate standards can be found in Table S2. Three of these identified compounds, tyrosine, tryptophan, and phenylalanine, were three of the 64 compounds upregulated by the presence of a BK neighbour (BK-BK/A) when compared to no neighbour (BK-0/A).

### Root morphology analysis

**Figure 5:**
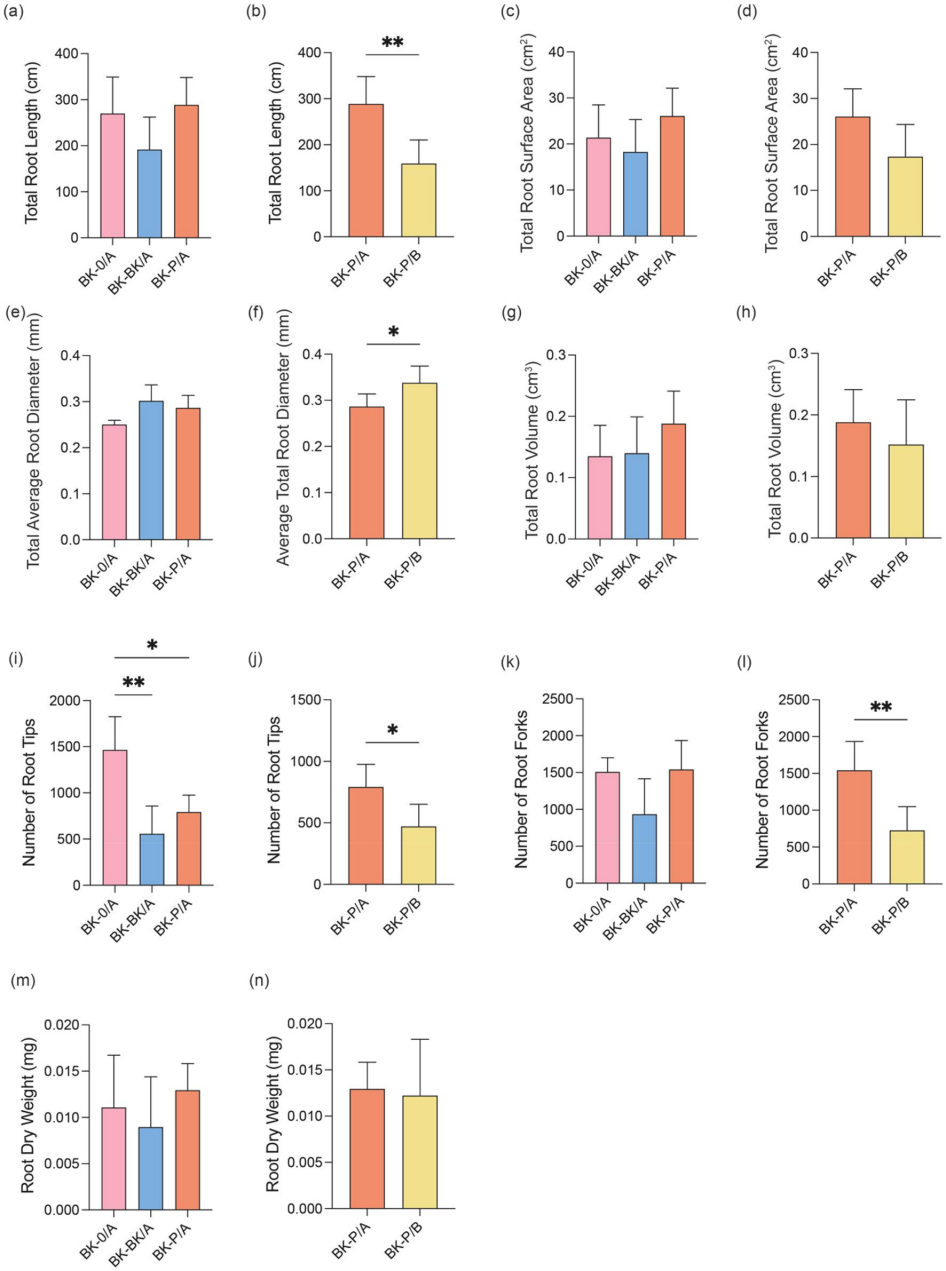
The impact of the presence of BK and P neighbours on root parameters of BK split-root. Comparison of BK root parameters between A compartments BK-0/A, BK-BK/A and BK-P/A (**a**,**c**,**e**,**g**,**i**,**k**,**m**) where there is no interaction between the roots of BK plants and the neighbours and comparison of BK root parameters between the two BK-P compartments BK-P/A with no root interaction between BK and P and (BK-P/B) with root interaction between BK and P (**b**,**d**,**f**,**h**,**j**,**l**,**n**). Total root length (**a**,**b**), Total root surface area (**c**,**d**), Average total root diameter (**e**,**f**), Total root volume (**g**,**h**), Number of root tips (**i**,**j**), Number of root forks (**k**,**l**), Root dry weight (**m**,**n**). The bars indicate the mean (n = 3–5), and error bars indicate the standard deviation. One-way ANOVA with Dunnett’s multiple comparison post hoc test was performed to determine significant differences in root parameters, 95% confidence level, p > 0.05 *p ≤ 0.05 **p ≤ 0.01 ***p ≤ 0.001.

Among the BK root parameters compared between the A compartments of split-root conditions (BK-0/A, BK-BK/A, and BK-P/A) (Fig. 5a,c,e,g,i,k,m), only the number of root tips decreased significantly in BK-BK/A and BK-P/A compared to BK-0/A. The number of root tips in BK-BK/A was lower than in BK-P/A.

When A and B compartments of BK-P were compared, significant differences were observed in multiple root parameters: total root length (Fig. 5b), number of root tips (Fig. 5j), and number of root forks (Fig. 5l) were significantly decreased, while the average total root diameter (Fig. 5f) was significantly increased in compartment B (BK-P/B) compared to compartment A (BK-P/A). The dry root weight was similar in both compartments (Fig. 5n). Even though the total root surface area was lower in BK-P/B compared to BK-P/A, the difference was not significant (Fig. 5d).

**Figure 6:**
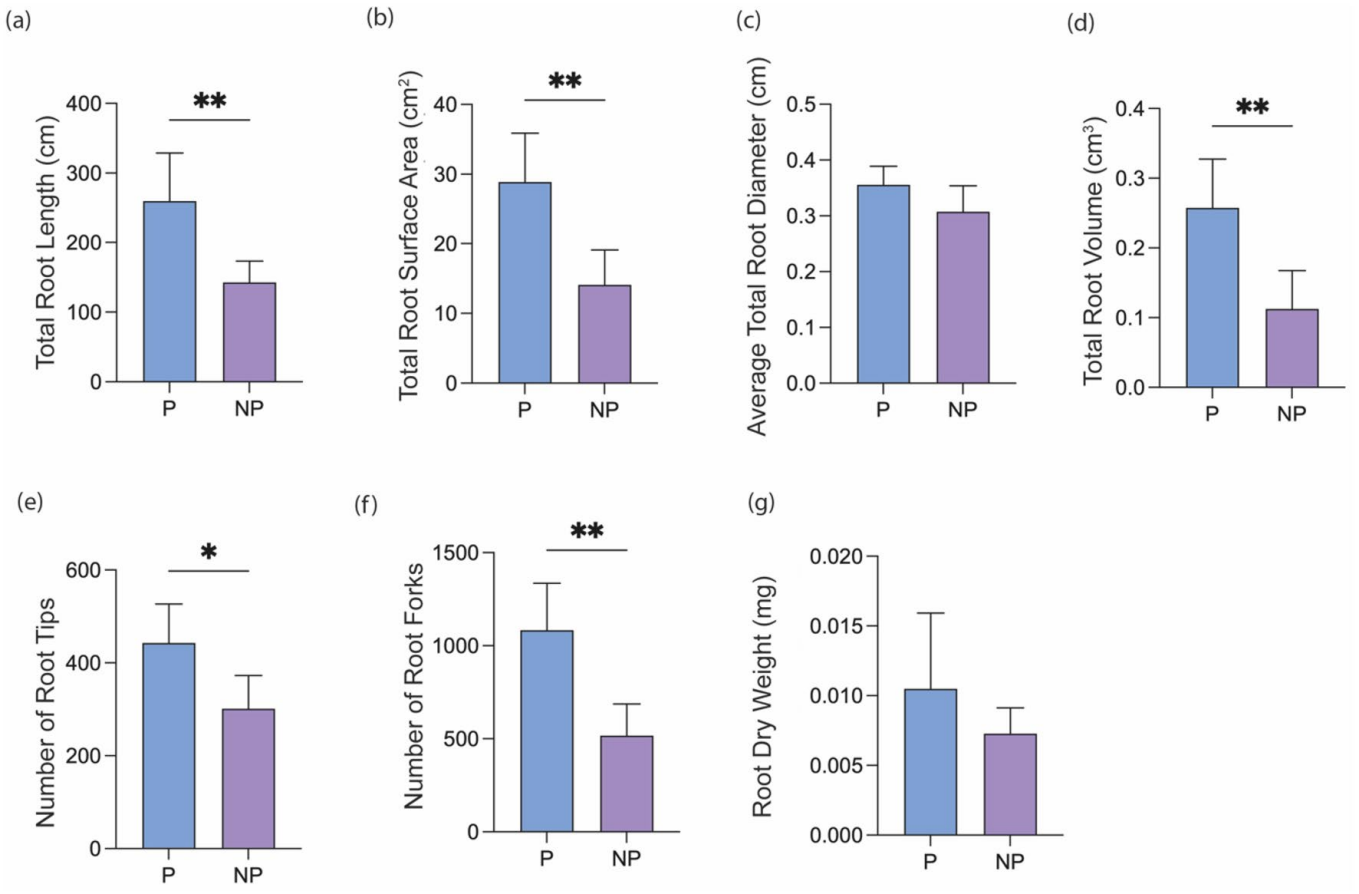
The effect of the presence of BK split-root on P root parameters. Total root length (**a**), total root surface area (**b**), Average total root diameter (**c**), Total root volume (**d**), Number of root tips (**e**), Number of root forks (**f**), Root dry weight (**g**) of P grown without a neighbour plant (P was compared to P grown as a neighbour to BK split-root (NP). The bars indicate the mean (n = 5), and the error bars indicate the standard deviation. One-way ANOVA with Dunnett’s multiple comparison post hoc test was performed to determine significant differences in root parameters. n = 5, 95% confidence level, p > 0.05 *p ≤ 0.05 **p ≤ 0.01 ***p ≤ 0.001.

When comparing the root parameters of P plants growing alone with those growing with split-root BK; neighbouring P (NP), a significant decrease was observed in total root length (Fig. 6a), root surface area (Fig. 6b), root volume (Fig. 6d), number of root tips (Fig. 6e), and number of root forks (Fig. 6f) of NP. Whereas there was no significant change in average total root diameter and root dry weight (Fig. 6c,g).

### BK root exudate treatment on P seedlings

**Figure 7:**
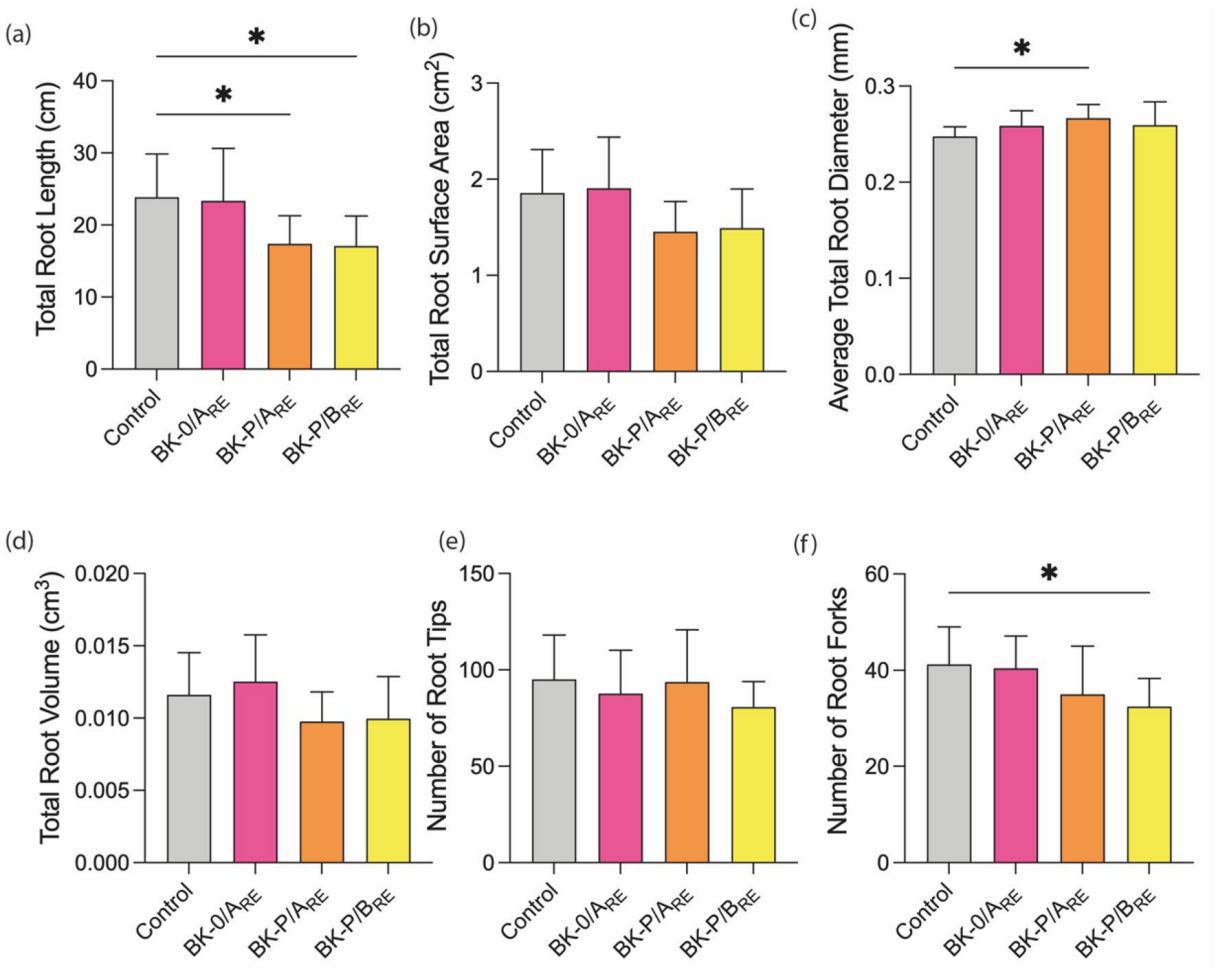
The impact of BK root exudate treatment on P root parameters. Total root length (**a**), Total root surface area (**b**), Average total root diameter (**c**), Total root volume (**d**), Number of root tips (**e**), Number of root forks (**f**) of P seedlings received root exudates obtained from the A compartments of BK split-root systems without neighbour (BK-0/A_RE_) and with P neighbours: having no direct root contact (BK-P/A_RE_), and a direct root contact (BK-P/B_RE_) and the controls received nano pure water. The bars indicate the mean (n = 9–10, each data point is an average of 4 P seedlings grown in the same cartridge), and error bars indicate the standard deviation. One-way ANOVA with Dunnett’s multiple comparison post hoc test was performed to determine significant differences in root parameters, 95% confidence level, p > 0.05 *p ≤ 0.05 **p ≤ 0.01 ***p ≤ 0.001.

P seedlings were treated with root exudates to evaluate the impact of root exudates derived from BK interacting with neighbouring plants on P root system architecture (Fig. 7a,b,c,d,e,f). The BK-P root exudate treatments (BK-P/A_RE_ and BK-P/B_RE_) significantly decreased total root length, while BK-P/B_RE_ treatment also significantly decreased the number of root forks (Fig. 7a,f), however treatment with BK-0/A root exudates (BK-0/A_RE_) exhibited no difference in the total root length of P seedlings compared to control plants. The average root diameter of P treated with BK-P/A_RE_ was significantly longer compared to control plants (Fig. 7c).

## Discussion

The study of plant root interactions presents challenges due to technical difficulties in accessing the root system without causing disturbance. While numerous studies utilize non-intrusive systems to study plant root systems, we established a novel split-root system and experimental design that, to our knowledge, is the first to address three major challenges all at once. This system (i) enabled root exudate collection without disturbing the root systems, (ii) allowed for the differential treatment of different parts of a single root system in separate compartments, (iii) and enabled the observation and characterization of changes in root exudate profiles of specific plants interacting with different neighbours. This allowed the investigation of the impact of the various factors in this study (i.e., BK response to different neighbours and P response to applications of exudates from the different neighbour setups) which returned novel insights into root exudation patterns in plant–plant interactions.

### BK root exudate metabolomics

In quantifying metabolomic changes in root exudate samples in response to the presence of a neighbouring plant using an NTA, discriminating the differences in individual metabolites under various tested conditions is crucial. Root exudation is a process that can be passive or active and is influenced by several environmental and biological factors. Therefore, finding a suitable biological normalization parameter presents challenges. Metabolites which are stably produced by the plant and relatively constant between different individuals and within an individual over time^66^ would be optimal markers for biological normalization. However, these types of markers are not always present and, in the absence of such markers for root exudate metabolomics, plant growth parameters are mainly used for normalisation. In our experiment, we observed weaker correlation between our overall metabolite signal and root dry weight when compared to the number of root tips (Fig. 2). This discrepancy is logical given that the spatial distribution pattern of root exudation is not homogeneous along the root axis. Root exudation is reported to be primarily occurring at the root tips^14^, resulting in localised effects in small, spatially distinct areas.

Utilizing the developed biological normalization method, root exudate metabolome analysis visualized species-related differences, separating P and BK root exudates samples (Fig. 3b). In a previous study, root exudate metabolomes of different plants species—*A. thaliana, B. distachyon* and *M. truncatula*—also differed from each other^64^. The authors speculated that plant root exudates share a core metabolome and that the presence of species-specific metabolites in exudates might distinctively regulate interactions in the soil. Similarly, the differences in the BK and P root exudate metabolome might change different parameters such as the dynamics of belowground crop–weed interactions and soil microbial composition^67^.

In this study, exudate differences are observed not only between species but also within buckwheat isolated compartments those grown alone (BK-0/A), those grown with another buckwheat neighbour (BK-BK/A), and those grown with a redroot pigweed neighbour (BK-P/A) (Figs. 3c, 4a–c), confirming our postulation that BK perceives the presence of intra- (BK) and interspecific (P) neighbours through interacting root systems and systematically modify its root exudate composition. Other studies have found similar responses. In a metabolomic study looking for metabolite changes in five plants species from the same community either growing alone or in association with one another, the authors concluded that, although the phylogenetic diversity of the neighbourhood did not have a strong effect on observed changes, the plants modulated their metabolic strategy to cope with the different levels of abiotic and biotic stress imposed by the presence of neighbours^68^. Specifically, in a rye hairy vetch co-culture, interspecific interaction affected root exudation of a flavonoid compound and, depending on the level of competition, the exudation of this flavonoid was increased or decreased^69^.

The particularity of our approach was the ability to look at systemic changes in root exudation. The observed differential expressions are likely to be attributed to long-distance internal signalling within BK from the B compartment to the A compartment. This process has been previously defined as “systemically induced root exudation of metabolites” (SIREM) and has been described in response to rhizosphere microorganisms^67^, nitrogen assimilation^70^, and nodule formation^69^. Specialized metabolites are mainly affected by SIREM^70^. In other studies, this signal has been observed to also induce metabolic changes in both leaves and roots. According to the literature, several candidates have been postulated as potential internal long-distance signalling molecules such as glycosylated forms of azelaic acid and pimelic acid and secreted peptides^70–72^ However, such mechanisms have not yet been characterized in BK.

Upon further looking into the specific compounds upregulated by the intraspecific or interspecific neighbours, there is limited general response to the presence of either neighbouring species tested as shown by the Venn diagram in Fig. 4d having only an overlap of ~ 7% of upregulated metabolites. It is evident that most changes in root exudates unique to the species tested as different neighbours trigger distinct responses. Similar findings were previously reported in another study where the accumulation and exudation of BK polyphenols differed depending on whether the neighbouring plant was *Lolium rigidum* Gaud. or *Portulaca oleracea* L^73^. We postulate that the difference in response could be for two reasons. Buckwheat may be recognizing other buckwheat or redroot pigweed neighbours (e.g., through the highlighted species-specific compounds) and responding to their unique cues. Species-specific soil chemical legacies also affect the vegetation composition and dynamics through plant-soil feedback effects^74^. Studies show that negative feedback in wheat result in the release of secondary metabolites of the family of the benzoxazinoids^75^. However, more information is needed to suggest that this is what is specifically happening, and further studies would be needed to assess persistence of metabolites within the substrate, the next generation of plants, and consequence of this possible feedback. Alternative or in addition to this specific chemical recognition, the different neighbours may be inducing different types of stress (resource competition, physical, and/or chemical) and the split buckwheat plant may be responding accordingly. A previous study supports this by showing that root exudates correlated with competitive traits (root respiration, N concentration) for 18 woody species^76^.

### Changes in BK root architecture

In analysing the effects of neighbouring plants on root system dynamics, the decrease observed in compartments where both BK and P neighbours were present without root-root contact (Fig. 5i) could be a part of the response to signals, as root tips improve the ability of plants to respond to both internal signals and external stimuli from the environment, explore the rhizosphere and absorb water and nutrients^77,78^. Root tips are the initial parts of the plant to explore new surroundings, they may influence the dynamics of plant–plant interactions. A previous study has shown that invasive grass species with a higher number of root tips have a greater chance of survival in field conditions^79^.

In our study, the decrease in the number of root tips in presence of a BK and P neighbour could be due to several factors: BK might have competed for essential resources and the root exudates play a role in the mobilization of soil nutrients, which in turn influences the architecture of the root system. In a previous study, the root exudate composition showed variation between different species and based on root morphology^80^. Alterations in root system architecture affect the root exudation, the attraction of microorganisms, as well as the function, decomposition, and fate of root exudates^81^. Therefore, rhizosphere microbial composition might be different, and/or the root exudates released from neighbouring plants might have had an inhibiting effect on BK root growth. In previous studies conducted on radish seedlings exposure to not root exudates but aqueous *Fallopia* extracts severely damaged the radish root tips and suppressed their growth^82,83^.

In our setup, despite P seedlings requiring less water, nutrients, and space due to their smaller stature compared to BK plants, they still lead to a significant decrease in the number of BK root tips. Furthermore, root parameters that are often associated with competition, such as total root length (which would provide an advantage in accessing resources if longer^84^) and total root surface area (providing a larger surface to uptake water and nutrients^85^) were not significantly different between compartments with and without a neighbour. This suggests that the reduction in root tips might not be explained solely by the competition for resources and that the composition of root exudates released by the neighbouring plant could play a role.

The fact that total root length, average root diameter, number of root tips, and number of root forks were lower in compartment with direct root-root contact than in compartment without (Fig. 5) indicates that BK preferred to allocate its roots in compartment which was not already occupied by another plant, and shifted the allocation of its roots in response to resource depletion or simply the presence of neighbouring plants. The roots of BK in the compartment where there is direct root-root contact sense the roots of neighbouring plants, water, and nutrient availability, subsequently generating local responses by triggering specific signalling pathways. In conclusion, not only did the root exudate profile change upon SIREM, but the BK root system architecture was also altered.

### Changes in the root architecture of P directly interacting with BK and in response to BK root exudate treatments

BK presence in split-root systems had a negative impact on the root growth of neighbour P as previously shown in Fig. 6. As previously discussed for BK, the changes in neighbouring P root parameters, in comparison to the root parameters of P grown alone, can be attributed to multiple factors such as resource competition between BK and P plants, spatial constraints, chemical compounds released from BK roots, and shade effects. The changes in P root growth, placement, and architecture by the presence of a neighbour plant is described sparsely in the literature. In our previous study, BK suppressed growth of P without physical root interactions^56^. In another study, the presence of maize significantly reduced the root density of P in shallow soil layers as the roots expanded into deeper soil in the presence of maize neighbour^86^. In a different study, while root density was affected, it was shown that wheat did not affect P root placement response. However, when 6-Methoxy-benzoxazolin-2-one, a wheat allelochemical, was applied, there was a response^87^.

Since plant–plant interactions involve a complex interplay between competition, chemical interactions, and adaptive responses that allow plants to survive and thrive. Controlling for as many environmental factors which impact plant vigour during the experiment(s) was pertinent. Specifically, when applying root exudates, it was essential to eliminate the effects of nutrient variations among different growing conditions. Also, most biological tests involve Petri dish assays where root exudates are applied only once to very young seedlings. When plants are grown together in split-root systems or under field conditions, they are exposed to compounds continuously released by the roots of every individual neighbour; this continual exposure might induce responses in each neighbouring plant. To mimic this exudate process without having a plant neighbour present to impact the physical space, BK root exudates were applied daily in small amounts (around 7.5% of the total volume extracted from half of a root system).

The root length, a parameter associated with the overall performance of a plant and its ability to occupy belowground space^88^, was reduced in P when treated with root exudates of BK interacting with P neighbours, both without direct root-root contact and with direct root contact (Fig. 7a). This agrees with our previous experiments^89^ where root derived compounds from BK when grown in close vicinity to P were applied to P seedlings which lead to decreased primary root elongation in comparison to root-derived compounds obtained from BK monocultures.

These findings suggest that changes in the chemical composition of BK root exudates induced by a P neighbour led to the total root length reduction of P seedlings. BK root exudates obtained from split-root systems interacting with P, irrespective of having direct root contact, resulting in local responses or no direct root contact, resulting in systemic responses, caused a significant decrease in total root length of P. On the other hand, the treatment of root exudates obtained from the compartment where the BK and P roots are in direct contact significantly decreased the number of root forks of P (Fig. 7f). We assume that among the 46 metabolites upregulated in BK in the presence of P, one or several of them are involved in the observed total root length reduction by BK-P interspecific interaction.

Moreover, we observed a higher reduction in the total root length of neighbour P in split-root systems than in the root exudates treatments. Additionally, various other P root parameters were significantly different in split-root systems in the presence of BK (Fig. 6). As anticipated, direct interaction between BK and P plants grown together cause more changes in root parameters of P. This might be due to competition between BK and P as well as a longer period of interaction (14 days of growth in split-root systems compared to 10 days of root exudate application). This could also be associated with improved allocation/diffusion/higher concentration of the root exudates when the plants are grown together than the root exudate application.

## Conclusion

This study demonstrates that root exudate metabolite profile of BK change in response to neighbouring plants and the root system and morphology of both BK and its neighbour P is impacted by their interaction. Moreover, the changes in root exudation are mostly unique to the neighbouring species tested. Furthermore, even when BK and P do not share the same space, root exudates obtained from co-culture still impact the P root system. By showing a direct link between physical presence, chemical exudation, and morphological response, these findings highlight the dynamic nature of root exudates and the implications of these cues on plants growing in proximity as a part of plant-plant communications. In future studies, gaining further insight by targeting the differentially expressed compounds of interest detected in this study to elucidate their chemical structure and genetics could pave the way for the development of new strategies in weed control. Once identified, these compounds could be purified and tested for their effects on different weed species. This would allow for the improvement of varieties or agricultural practices to increase the concentration and persistence of weed suppressing compounds in cover crop exudates to maximise the effect of these exudates on weed community.

## Material and methods

### Plant material and growth conditions

Square 120 × 15 mm Petri dishes (Corning) were lined with two sheets of autoclaved Whatman filter papers and moistened using half-strength Hoagland’s solution (Hoagland's No. 2 Basal Salt Mixture, Sigma-Aldrich) with a pH of 5.8. A row of five BK (variety: Lileja) seeds were placed in each Petri dish and covered with half-sized filter papers. They were sealed using parafilm and placed on a stand at a 90° angle in a phytotron (Aralab, Clitec) set at 24 °C for 16 h and 18 °C for 8 h with a relative humidity of 70% and kept in the dark for 3 days.

Emerging BK roots were carefully cut a few millimetres above the root apex using a sterilised scalpel (Fig. 1a) to induce the development of secondary roots^90^ facilitating the subsequent splitting of the root system (Fig. 1c). Root cutting is a stress-inducing procedure that requires a recovery process. Partial root cutting with 2 weeks of recovery was chosen because it induces less stress than de-rooting^91^. The Petri dishes were again sealed with parafilm and transferred to the phytotron set at, 16:8-h light/dark photoperiod, 24/18 °C and a relative humidity of 70% for 5 days. The 7-day-old BK seedlings were transferred to split-root systems (Fig. 1b).

### Split-root system preparation

All plants were grown in 60 mL solid-phase extraction (SPE) (Bond Elut 12131018, Agilent Technologies) filled with 250–400 µm glass beads (Guyson SA). The outside of the cartridges were covered with black self-adhesive film (Fig. 1e). For split-root systems, two SPE cartridges were modified by melting a small semi-circle at their tops to stabilise the seedlings and then they were affixed together. For non-split-root conditions, a single SPE cartridge was filled with glass beads. BK seedlings were carefully transferred from Petri dishes. The roots were counted for each seedling and divided into two equal parts; the seedlings with an odd number of roots were balanced by placing the longest root into the compartment with fewer roots (Fig. 1c). The divided roots were placed in the SPE cartridges to create two compartments, which will be called compartment A and B onward. After splitting the BK roots, neighbour plant seeds were only placed within compartment B. Neighbours were either BK seeds for homospecific interactions or P seeds for heterospecific interactions. P seeds were harvested from a field at Agroscope, Changins, Switzerland and the use P plants in the present study complies with international, national and institutional guidelines and the study was conducted in accordance with relevant legislation. This established three split-root system conditions, each with two compartments, as well as two non-split-root conditions and blank controls (Fig. 1d). Each cartridge filled with glass beads was moistened with half-strength Hoagland's solution. Initially, two BK and ten P seeds were sown in their respective B compartments. After germination, BK seedlings were thinned down to one and P seedlings to three. Plants were grown in a phytotron with a 16:8-h light/dark photoperiod at 24/18 °C and 70% relative humidity for 2 weeks. Each compartment was irrigated with 5 ml of half-strength Hoagland's solution daily, including blanks with no plants. Root exudate extraction was done on the 14th day (n = 3–5).

### Manifold setup and root exudate extraction

Root exudates were collected from split and non-split-root plants grown in SPE cartridges. Exudates were extracted using a SPE vacuum manifold (Macherey–Nagel) connected to a vacuum pump (V-300, Buchi) controlled by a Buchi I-300 Pro Interface. Conical centrifuge tubes, 50 mL (Corning) were positioned under stainless-steel needles (Macherey–Nagel). The interface to the pump was set at 780 mbar, maintaining the glass chamber pressure at 5 mmHg. Root exudates were collected from (i) compartment A with no direct root contact: split-root BK without a neighbour (BK-0/A), split-root BK with neighbouring BK (BK-BK/A) and split-root BK with neighbouring P (BK-P/A), (ii) compartment B with direct root contact: split-root BK with neighbouring P (BK-P/B), and iii) non-split-root BK and P, and blank controls (Fig. 1d and e).

### Root exudate collection for mass spectrometry analysis

SPE cartridges were placed on the stopcock valves of the manifold. The vacuum pump was started, and 30 ml extraction solvent was added to each cartridge over the span of 30 s. Extraction solvent consisted of 95% (w/v) methanol (Merck Uvasol), 4.95% (w/v) nano pure water, and 0.05% (w/v) formic acid (VWR, HiPerSolv Chromanorm for LC–MS) with internal standard 3,5-di-tert-butyl-4-hydroxybenzoic acid (Sigma-Aldrich) added to a final concentration of 0.5 µmol L^−1^. The stopcock valves were left open for an additional 30 s, making it so root exudates were vacuumed off for a total of 1 min per sample to minimise root exposure to the extraction solvent. From each sample, 10 ml was transferred to 16 × 160 mm Pyrex test tubes (SciLabware) and evaporated using a sample vacuum concentrator (Genevac EZ-2 Plus) set at 35 °C. The concentrator was initially set to “HPLC mode” for an hour, followed by an “aqueous mode” for 2 h or longer if extra drying was required to achieve a final approximate volume of 200 µl to ensure that the samples were not dried to completion.

### Root exudate collection for application on P

Root exudates were collected from split-root systems using the manifold setup mentioned previously. However, this time nano pure water was used as an extraction solvent. 30 ml of nano pure water was added for 30 s to each cartridge and vacuumed for one minute to wash off the Hoagland’s solution. Then, 15 ml of nano pure water was added to each cartridge and plants were re-placed in the phytotron for 24 h to allow for regeneration and release of root exudates. Root exudates were then again extracted with 30 ml of nano pure water. This resulted in a second set of root exudates which did not vary in Hoagland’s salt concentrations across different experimental conditions. The matrix consistency of the extracts was confirmed by measuring electrical conductivity and pH. Second extracts obtained following the 24 h incubation were pooled together to obtain a homogeneous mixture. Ten P seeds were sown in single SPE cartridges. Three millilitres of half-strength Hoagland’s solution were added to each tube every day for the first 5 days. The P seedlings were thinned down to four plants on the fifth day. Over the next 10 days, the P plants were given 3 ml of BK-0, BK-P/A, or BK-P/B root exudates, and controls were given 3 ml of nano pure water every day. They were also given 1 ml of half-strength Hoagland’s solution every other day to make sure that they had enough nutrients (n = 10). At the end of the treatment, root morphology analysis was performed on P seedlings.

### Root sample preparation, digitising and analysing roots

Plants were gently removed from their cartridges and thoroughly washed to eliminate glass beads after both the collection of root exudates and root exudate application. Root samples were transferred to a transparent tray (Reagent Instruments Inc.) and submerged in water. They were carefully spread out to minimise overlapping and scanned using a scanner (Epson Perfection V700 Photo). Root parameters: total root length (mm), root surface area (mm^2^), average total root diameter (mm), root volume (mm^3^), and number of root tips and forks were analysed using WinRHIZO Basic 2021 (Regent Instruments Inc.). Also, both aboveground and root dry weight (mg) was measured by placing them in a drying oven at 50 °C for 48 h then weighing them. Statistical significance (p < 0.05) of root parameters were assessed by either a student's t-test or one-way ANOVA followed by post hoc Dunnett's multiple comparisons test considering BK-0/A as the control and comparing each mean against this control using GraphPad Prism (version 9.2.0 for MacOS, GraphPad Software).

### Sample preparation and non-targeted mass spectrometric analysis

Concentrated sample extracts (~ 200 µl) were transferred to 1.5 mL HPLC amber glass vials (Altmann Analytik) and reconstituted to a 30-fold concentration of the original volume in 89.9% ultrapure H2O (MilliQ with an LC-Pak attachment, Merck), 10% methanol (CHROMASOLV, Honeywell), and 0.1% formic acid (% v/v, Fluka, Honeywell). Particulate was removed using an Amicon 30 kDa Ultra-0.5 Centrifugal Filter (Merck). Quality control (QC) samples comprised of a pooled sample. Samples, including QCs, were separated into two aliquots of 100 µL for both negative and positive ionization mode and stored at -80 °C until measurement and then stored at 4 °C at the autosampler (1290 Infinity II, Agilent Technologies) during the measurement sequence. The liquid chromatography quadrupole time-of-flight (LC-QTOF-MS) system was controlled by MassHunter acquisition software (version 10.1, Agilent Technologies). Samples were injected in randomized order. QCs were measured after every 13th or 14th sample to monitor the performance of the LC-QTOF-MS system and normalize data. An UHPLC system with binary pump (1290 Infinity II, Agilent Technologies) was used to perform chromatographic separation on a Discovery HS F5 (150 × 2.1 mm, 3 μm particle size, Sigma-Aldrich) pentafluorophenyl column paired with a Discovery HS F5 Supelguard Cartridge (Sigma-Aldrich). Mobile phase A was ultrapure water with 0.1% formic acid (% v/v, Fluka, Honeywell) and mobile phase B was methanol (CHROMASOLV, Honeywell) with 0.1% formic acid (% v/v). The gradient is shown in Table S3a. Injection volume was 5 μL. The total analysis time was 15.5 min per injection, with a constant flow rate of 350 μL min^−1^ and 50 °C column oven (1290 Infinity II) temperature. Reference mass solution (HP-0921, Purine, TFANH_4_, Agilent Technologies) was pumped (1260 Infinity, Agilent Technologies) in tandem via a secondary sprayer to the electrospray ionization interface for online mass calibration (m/z = 121.0509 and 922.0098 (+); m/z = 119.0363 and 966.0007 (−)).

Data was collected with an LC-QTOF-MS (6560, Agilent Technologies, Santa Clara, CA, USA) with a Dual AJS ESI source. Data was acquired in data dependent acquisition (DDA) mode with a mass to charge ratio (m/z) range of 50 to 1700 and five max precursors per cycle and stored in profile mode. Source parameters and more detailed data acquisition information can be found in Table S3b.

A mock root exudate mixture was made up of 58 chemical standards of compounds known from literature to be exuded by the roots of plants (Table S4). This was used to check the repeatability of the signal intensity and retention times of the instrument.

### Non-targeted LC–MS/MS data evaluation

After data acquisition, data was reprocessed with the Agilent reprocessing software (MassHunter Workstation version 10.0, Agilent Technologies) by centroiding data and recalibrating to the reference solution.

Pre-processing was done using MS DIAL (version 4.9^92^). Only peaks which eluted past the retention time lower cutoff of 1.8 min with a minimum height of 1E3 and a minimum peak width of 6 data points were picked. Isotopologues were aggregated and adducts were annotated. An in-house database was applied for identity confirmation which matched features based upon their retention time, m/z, and MS2 fragmentation pattern. This database was generated using data from the aforementioned mock root exudate mixture in MS FINDER, version 3.6 These metabolites were identified to a level 1 according to Schymanski et al.^65^. Picked peaks were then aligned across samples with an MS1 tolerance 15 mDa and a retention time tolerance of 0.2 min. After alignment, gap filling of missing values was performed. Further blank (fivefold sample average/blank average) and minimum of 60% group presence filtration was applied. Data for all compounds was normalised to the internal standard and to pooled QC samples using locally weighted scatterplot smoothing (LOWESS) regression. More detailed MS DIAL pre-processing parameters can be found in Table S5.

Further data manipulation and statistical analysis was performed in RStudio (Version 2023.06.0+421). An average signal-to-noise (S/N) cut-off of 10 was applied. Adducts which did not have a corresponding protonated or deprotonated value were re-annotated as M+H or M−H features. Adducts were matched to their protonated or deprotonated counterparts and peak area values were added together to generate a total compound signal (TCS) value. For finding a suitable parameter for biological normalization, TCS values were correlated to either the number of root tips or root weight in grams and finally normalised by dividing TCS by the number of root tips. For BK-P/B (see Fig. 1d), there were compounds exuding from both BK and P roots. The exuded metabolite mixture made it not possible to biologically normalise to a single species for most metabolites. However, if a compound was specific to a single species, the value was biologically normalised to the species that metabolite was unique to in compartment BK-P/B. Since QC samples were generated from a pooled sample, they were normalised to the average number of root tips per compartment.

For each sample, the individual TCS values were added together to reduce the data into one cumulative variable. Additionally, each sample still had hundreds of individual TCS variables for each compound. Both the cumulative TCS values and each individual TCS signals underwent univariate statistical assessment. The means, distributions, and variation were calculated which were visualised through the creation of box and whisker plots. Outliers were defined and calculated as anything that is more than 1.5 * interquartile range (IQR) above the third quartile or below the first quartile. Among group variation was assessed through *F*-testing when two groups were compared or Bartlett’s test for multiple groups. Significance testing was performed using a t-test when comparing two groups or an ANOVA when comparing multiple. When comparing groups with unequal variance, both t-test and ANOVA were modified to Welch’s variants. Either a Dunnett’s T3 post hoc test modified to allow for a control group (this was calculated using GraphPad Prism version 9.2.0) or a Games-Howell post hoc test were calculated depending on the comparisons being made. Significant metabolites of interest were visualised through creation of volcano plots which utilized aforementioned Welch’s t-test in addition to fold change calculations.

Multivariate dimension reduction was performed using each individual TCS as an input variable and visualised through principal component analysis (PCA) and partial least square discriminant analysis (PLS-DA). All data was centred and auto scaled before multivariate analysis.

### Supplementary Information

Supplementary Information.

## Data Availability

The datasets used and/or analysed during the current study available from the corresponding author on reasonable request.
