# Supplementary material for: Neighbour-induced changes in root exudation patterns of buckwheat results in altered root architecture of redroot pigweed
Source: Sci Rep. 2024 Apr 15;14:8679. doi: 10.1038/s41598-024-58687-3 (PMC11018816; doi:10.1038/s41598-024-58687-3)
Supplement: Supplementary file 1 — Supplementary Information. [file 41598_2024_58687_MOESM1_ESM.docx]

**Supplementary Information**

**Neighbour-induced changes in root exudation patterns of buckwheat results in altered root architecture of redroot pigweed**

Çağla Görkem Eroğlu 1,a , Alexandra A. Bennett 2,a, Teresa Steininger-Mairinger 2, Stephan Hann 2, Markus Puschenreiter 3, Judith Wirth 1, Aurélie Gfeller 1

1 Herbology in Field Crops, Plant Production Systems, Agroscope, Nyon, Switzerland

2 Department of Chemistry, Institute of Analytical Chemistry, University of Natural Resources and Life Sciences, Vienna (BOKU), 1190, Vienna, Austria

3 Department of Forest and Soil Sciences, Institute of Soil Research, Rhizosphere Ecology & Biogeochemistry Group, University of Natural Resources and Life Sciences, Vienna, Konrad-Lorenz-Strasse 24, 3430, Tulln, Austria

[Figure S1: Box plots showing differences in normalised cumulative total compound signal (TCS) in both positive and negative ionisation modes. TCS was calculated by summing the areas of adduct and non-adduct peaks resulting from the same compound. All signals were added together within a single sample to create a cumulative value to represent total metabolite concentration. Data was normalised to internal standard (3,5-Di-tert-butyl-4-hydroxybenzoic acid) and pooled QC samples using the LOWESS method. *n* = 3-5/condition. Box boundaries indicate the 25^th^ to the 75^th^ percentile with median represented by lines. Whiskers are min to max data points. Outliers are labelled with their replicate number. When looking at data that has not been biologically normalised and comparing buckwheat non split samples (BK) to split root buckwheat (BK-0/A), BK samples are lower (significantly so for positive ionisation mode only) in overall metabolite signal compared to BK-0/A. Comparatively, data that has been biologically normalised to the number of root tips has more equal variance and no significant (p > 0.05) difference in total signal when comparing BK to BK-0/A (b). 3](#_Toc159947364)

[Figure S2: Box plot of selected compounds expressed in either buckwheat only (a) or redroot pigweed only (b). For compounds which are found in buckwheat only (unknown metabolite 813, ionisation mode = positive, m/z = 327.1049, RT = 10.45), data from the shared compartment of the buckwheat split root setup where redroot pigweed is the neighbour (BK-P/B) is normalised to the number of buckwheat root tips. Conversely, redroot pigweed only compounds (unknown metabolite 341, ionisation mode = positive, m/z = 203.0810, RT = 4.02) are normalised to the number of redroot pigweed root tips. *n* = 3-5/condition. Box boundaries indicate the 25th to the 75th percentile with median represented by lines. Whiskers are min to max data points. A Games-Howell post-hoc test was performed to assess significance (p < 0.05) and generate connected letter values shown. 4](#_Toc159947365)

[Table S1: Differential expression of metabolites and their M/z and retention times (RT) are shown. Compounds were considered significantly different between groups if the total compound signal (TCS) false discovery rate (FRD) adjust p-value for a Welch’s t-test was <0.05 and if the Log_2_ fold change was >0.06 or <-0.06. Comparisons of compartment A of buckwheat samples grown in a split root setup with no neighbour (BK-0/A) versus another homospecific buckwheat neighbour (BK-BK/A) (a), (BK-0/A) versus buckwheat grown with a heterospecific pigweed neighbour (BK-P/A) (b), and for homospecific neighbour (BK-BK/A) versus a heterospecific neighbour (BK-P/A) (c) were performed. 5](#_Toc159947366)

[Table S2: Compounds identified to confidence level 1 according to the Schymanski scale^1^ in buckwheat samples and the retention times and m/z values are listed. While the entire fragmentation pattern of each compound was used for identification, confirmation fragment 1 and 2 were pulled from the fragmentation pattern to show validation of confirmation. 11](#_Toc159947367)

[Table S3: Parameters for LC-HRMSMS data acquisition showing the gradient of the liquid chromatography method (a) and the other chromatographic and mass spectrometric conditions (b). 12](#_Toc159947368)

[Table S4: The mock root exudate mixture was made up of 60 chemical analytical standards of compounds known from literature to be exuded by the roots of plants. Species which were found to exudate each compound and the citation for that species is given in the species column. Full reference information for each species citation can be found in the references section of this supplementary file. The company which sourced each standard is also given. 14](#_Toc159947369)

[Table S5: MS DIAL pre-processing parameters 20](#_Toc159947370)

[References 22](#_Toc159947371)


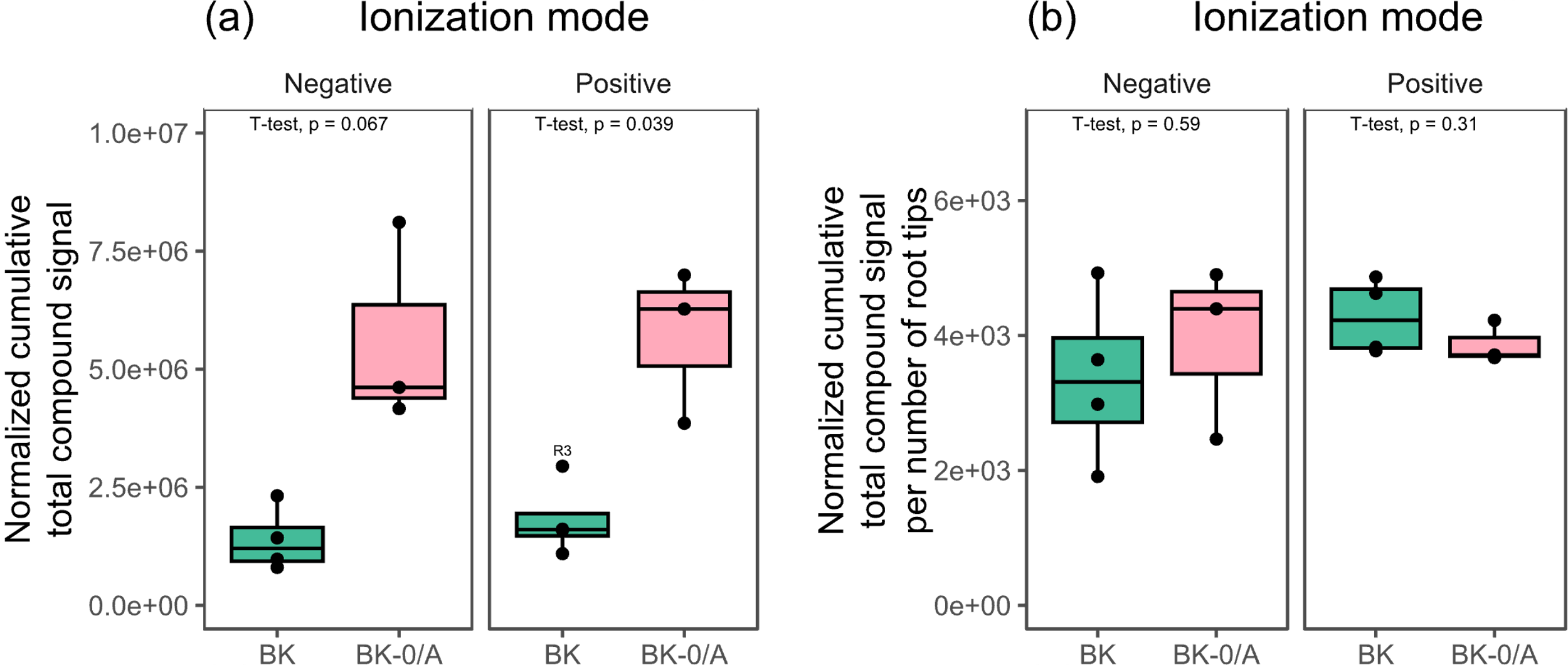


# Figure S1: Box plots showing differences in normalised cumulative total compound signal (TCS) in both positive and negative ionisation modes. TCS was calculated by summing the areas of adduct and non-adduct peaks resulting from the same compound. All signals were added together within a single sample to create a cumulative value to represent total metabolite concentration. Data was normalised to internal standard (3,5-Di-tert-butyl-4-hydroxybenzoic acid) and pooled QC samples using the LOWESS method. *n* = 3-5/condition. Box boundaries indicate the 25^th^ to the 75^th^ percentile with median represented by lines. Whiskers are min to max data points. Outliers are labelled with their replicate number. When looking at data that has not been biologically normalised and comparing buckwheat non split samples (BK) to split root buckwheat (BK-0/A), BK samples are lower (significantly so for positive ionisation mode only) in overall metabolite signal compared to BK-0/A. Comparatively, data that has been biologically normalised to the number of root tips has more equal variance and no significant (p > 0.05) difference in total signal when comparing BK to BK-0/A (b).

**
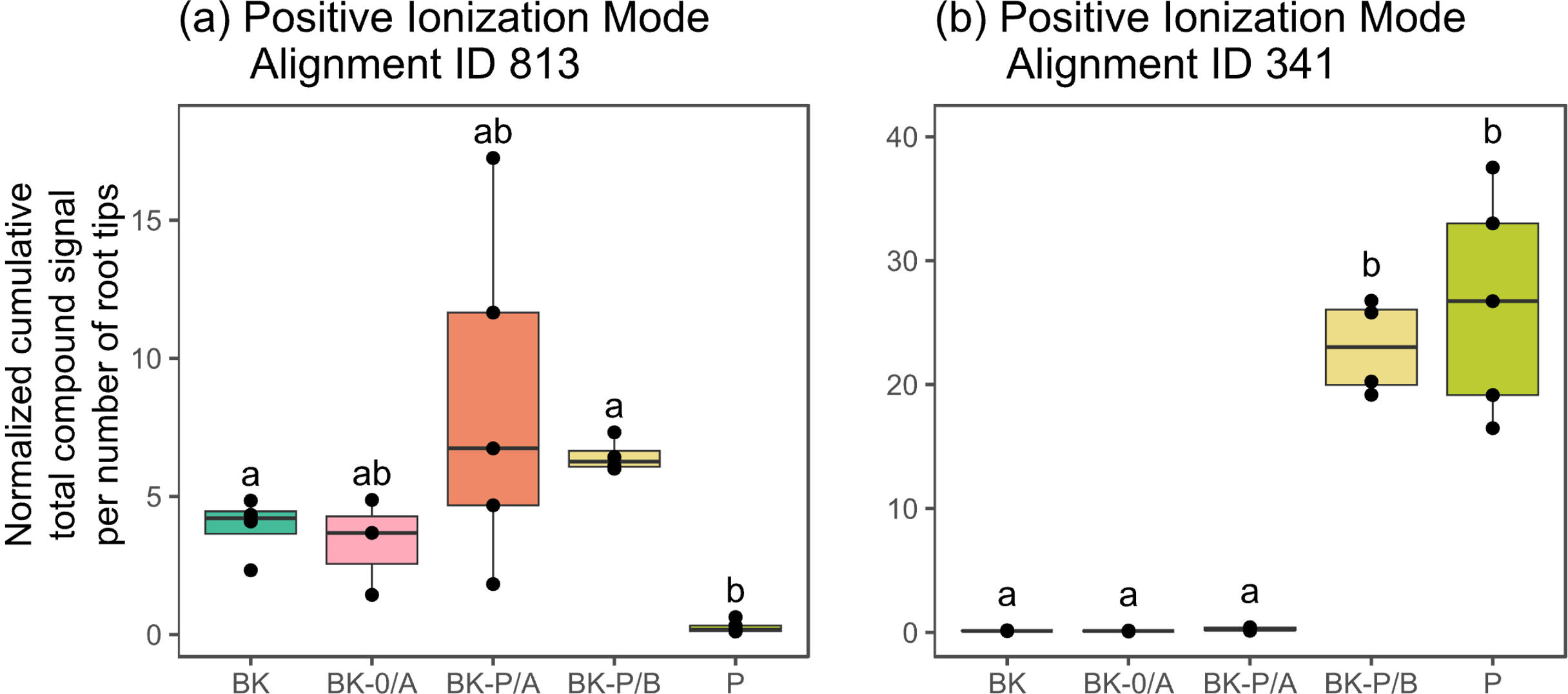
**

# Figure S2: Box plot of selected compounds expressed in either buckwheat only (a) or redroot pigweed only (b). For compounds which are found in buckwheat only (unknown metabolite 813, ionisation mode = positive, m/z = 327.1049, RT = 10.45), data from the shared compartment of the buckwheat split root setup where redroot pigweed is the neighbour (BK-P/B) is normalised to the number of buckwheat root tips. Conversely, redroot pigweed only compounds (unknown metabolite 341, ionisation mode = positive, m/z = 203.0810, RT = 4.02) are normalised to the number of redroot pigweed root tips. *n* = 3-5/condition. Box boundaries indicate the 25th to the 75th percentile with median represented by lines. Whiskers are min to max data points. A Games-Howell post-hoc test was performed to assess significance (p < 0.05) and generate connected letter values shown.

# Table S1: Differential expression of metabolites and their M/z and retention times (RT) are shown. Compounds were considered significantly different between groups if the total compound signal (TCS) false discovery rate (FRD) adjust p-value for a Welch’s t-test was <0.05 and if the Log_2_ fold change was >0.06 or <-0.06. Comparisons of compartment A of buckwheat samples grown in a split root setup with no neighbour (BK-0/A) versus another homospecific buckwheat neighbour (BK-BK/A) (a), (BK-0/A) versus buckwheat grown with a heterospecific pigweed neighbour (BK-P/A) (b), and for homospecific neighbour (BK-BK/A) versus a heterospecific neighbour (BK-P/A) (c) were performed.

(a)

| Alignment ID | Ionization Mode | Metabolite name | Average RT (min) | Average M/z | BK-0/A TCS | BK-BK/A TCS | FDR adjusted  p-value | Log_2_ fold change | Upregulated in |
| --- | --- | --- | --- | --- | --- | --- | --- | --- | --- |
| 57 | Positive | Unknown | 6.58 | 103.05 | 54.91 | 180.52 | 0.02 | 1.72 | BK-BK/A |
| 80 | Positive | Unknown | 3.91 | 119.05 | 0.90 | 4.02 | 0.03 | 2.16 | BK-BK/A |
| 95 | Positive | Unknown | 3.92 | 123.04 | 1.34 | 5.40 | 0.04 | 2.01 | BK-BK/A |
| 114 | Positive | Unknown | 8.27 | 132.08 | 1.15 | 6.55 | 0.02 | 2.51 | BK-BK/A |
| 127 | Positive | Unknown | 3.92 | 136.08 | 2.80 | 11.34 | 0.04 | 2.02 | BK-BK/A |
| 151 | Positive | Unknown | 8.26 | 143.07 | 0.36 | 2.11 | 0.05 | 2.57 | BK-BK/A |
| 156 | Positive | Unknown | 8.27 | 144.08 | 1.97 | 12.00 | 0.01 | 2.61 | BK-BK/A |
| 160 | Positive | Unknown | 8.27 | 146.06 | 7.10 | 43.31 | 0.01 | 2.61 | BK-BK/A |
| 210 | Positive | Unknown | 8.27 | 159.09 | 1.59 | 10.29 | 0.01 | 2.69 | BK-BK/A |
| 213 | Positive | Unknown | 8.27 | 160.08 | 0.22 | 1.53 | 0.02 | 2.78 | BK-BK/A |
| 94 | Negative | Phenylalanine | 6.56 | 164.07 | 1.80 | 10.90 | 0.05 | 2.60 | BK-BK/A |
| 247 | Positive | Unknown | 8.27 | 170.06 | 1.26 | 6.83 | 0.02 | 2.44 | BK-BK/A |
| 121  278 | Negative  Positive | Tyrosine | 3.91 | 180.07  182.08 | 0.61  5.28 | 5.10  21.61 | 0.04  0.04 | 3.06  2.03 | BK-BK/A |
| 341 | Positive | Unknown | 4.02 | 203.08 | 0.12 | 0.66 | 0.01 | 2.51 | BK-BK/A |
| 180  347 | Negative  Positive | Tryptophan | 8.25 | 203.08  205.10 | 7.83  72.98 | 71.51  455.86 | 0.02  0.02 | 3.19  2.64 | BK-BK/A |
| 342 | Positive | Unknown | 2.07 | 203.15 | 1.17 | 3.25 | 0.04 | 1.48 | BK-BK/A |
| 215 | Negative | Unknown | 2.58 | 216.05 | 0.21 | 1.41 | 0.05 | 2.73 | BK-BK/A |
| 402 | Positive | Unknown | 6.40 | 220.12 | 21.13 | 52.60 | 0.04 | 1.32 | BK-BK/A |
| 403 | Positive | Unknown | 4.03 | 221.09 | 0.09 | 0.77 | 0.02 | 3.07 | BK-BK/A |
| 489 | Positive | Unknown | 10.56 | 243.09 | 10.18 | 32.24 | 0.02 | 1.66 | BK-BK/A |
| 321 | Negative | Unknown | 8.21 | 250.07 | 3.39 | 20.74 | 0.05 | 2.61 | BK-BK/A |
| 352 | Negative | Unknown | 11.45 | 259.19 | 3.53 | 11.45 | 0.03 | 1.70 | BK-BK/A |
| 397 | Negative | Unknown | 11.57 | 273.21 | 4.07 | 11.24 | 0.04 | 1.46 | BK-BK/A |
| 704 | Positive | Unknown | 7.53 | 295.12 | 2.97 | 9.55 | 0.03 | 1.69 | BK-BK/A |
| 785 | Positive | Unknown | 6.73 | 319.14 | 1.28 | 2.61 | 0.05 | 1.02 | BK-BK/A |
| 527 | Negative | Unknown | 11.68 | 323.22 | 0.24 | 0.86 | 0.04 | 1.85 | BK-BK/A |
| 801 | Positive | Unknown | 8.39 | 324.23 | 0.07 | 0.35 | 0.04 | 2.25 | BK-BK/A |
| 828 | Positive | Unknown | 11.37 | 331.19 | 1.69 | 6.28 | 0.05 | 1.90 | BK-BK/A |
| 831 | Positive | Unknown | 4.89 | 332.13 | 3.58 | 12.10 | 0.05 | 1.76 | BK-BK/A |
| 590 | Negative | Unknown | 8.19 | 348.04 | 0.16 | 1.13 | 0.05 | 2.82 | BK-BK/A |
| 597 | Negative | Unknown | 8.02 | 351.03 | 0.17 | 0.77 | 0.04 | 2.14 | BK-BK/A |
| 612 | Negative | Unknown | 7.52 | 355.10 | 0.84 | 4.36 | 0.03 | 2.38 | BK-BK/A |
| 911 | Positive | Unknown | 7.91 | 359.14 | 0.34 | 1.19 | 0.04 | 1.82 | BK-BK/A |
| 661 | Negative | Unknown | 7.33 | 373.12 | 1.25 | 10.30 | 0.05 | 3.04 | BK-BK/A |
| 668 | Negative | Unknown | 11.05 | 375.18 | 0.56 | 1.60 | 0.05 | 1.53 | BK-BK/A |
| 678 | Negative | Unknown | 6.71 | 379.03 | 0.15 | 0.68 | 0.02 | 2.17 | BK-BK/A |
| 969 | Positive | Unknown | 7.91 | 381.12 | 0.06 | 0.29 | 0.02 | 2.38 | BK-BK/A |
| 708 | Negative | Unknown | 6.68 | 393.09 | 0.16 | 0.54 | 0.05 | 1.81 | BK-BK/A |
| 737 | Negative | Unknown | 7.32 | 403.08 | 0.07 | 0.52 | 0.04 | 2.81 | BK-BK/A |
| 1037 | Positive | Unknown | 7.26 | 403.16 | 1.12 | 3.15 | 0.02 | 1.50 | BK-BK/A |
| 1054 | Positive | Unknown | 7.49 | 411.16 | 0.32 | 1.21 | 0.03 | 1.94 | BK-BK/A |
| 764 | Negative | Unknown | 6.81 | 418.04 | 0.13 | 0.53 | 0.04 | 2.08 | BK-BK/A |
| 1102 | Positive | Unknown | 7.53 | 427.16 | 2.82 | 7.14 | 0.04 | 1.34 | BK-BK/A |
| 848 | Negative | Unknown | 6.79 | 455.18 | 1.73 | 4.48 | 0.02 | 1.37 | BK-BK/A |
| 853 | Negative | Unknown | 10.60 | 457.17 | 0.16 | 0.41 | 0.02 | 1.36 | BK-BK/A |
| 855 | Negative | Unknown | 8.20 | 459.15 | 1.30 | 6.75 | 0.00 | 2.37 | BK-BK/A |
| 873 | Negative | Unknown | 6.84 | 469.07 | 0.14 | 0.66 | 0.04 | 2.27 | BK-BK/A |
| 875 | Negative | Unknown | 7.13 | 469.19 | 5.08 | 15.38 | 0.02 | 1.60 | BK-BK/A |
| 878 | Negative | Unknown | 6.75 | 471.08 | 0.11 | 0.47 | 0.04 | 2.16 | BK-BK/A |
| 1279 | Positive | Unknown | 7.22 | 487.21 | 1.07 | 3.89 | 0.01 | 1.86 | BK-BK/A |
| 1300 | Positive | Unknown | 7.15 | 493.19 | 0.86 | 2.29 | 0.01 | 1.42 | BK-BK/A |
| 929 | Negative | Unknown | 6.87 | 497.06 | 0.06 | 0.27 | 0.03 | 2.06 | BK-BK/A |
| 934 | Negative | Unknown | 11.08 | 498.21 | 0.08 | 0.39 | 0.04 | 2.24 | BK-BK/A |
| 1327 | Positive | Unknown | 10.48 | 504.34 | 1.49 | 4.95 | 0.04 | 1.74 | BK-BK/A |
| 955 | Negative | Unknown | 7.22 | 509.22 | 5.32 | 25.34 | 0.02 | 2.25 | BK-BK/A |
| 967 | Negative | Unknown | 6.89 | 515.09 | 1.64 | 5.12 | 0.05 | 1.65 | BK-BK/A |
| 1357 | Positive | Unknown | 10.84 | 518.35 | 1.25 | 4.00 | 0.03 | 1.68 | BK-BK/A |
| 989 | Negative | Unknown | 6.71 | 531.10 | 0.10 | 0.35 | 0.04 | 1.76 | BK-BK/A |
| 995 | Negative | Unknown | 8.15 | 534.12 | 0.00 | 0.11 | 0.02 | 4.50 | BK-BK/A |
| 1026 | Negative | Unknown | 7.22 | 561.19 | 1.38 | 5.89 | 0.05 | 2.10 | BK-BK/A |
| 1035 | Negative | Unknown | 6.65 | 571.10 | 0.02 | 0.18 | 0.02 | 3.15 | BK-BK/A |
| 1492 | Positive | Unknown | 6.71 | 608.17 | 0.06 | 0.21 | 0.03 | 1.91 | BK-BK/A |
| 1509 | Positive | Unknown | 10.39 | 618.18 | 0.02 | 0.06 | 0.01 | 1.91 | BK-BK/A |
| 1184 | Negative | Unknown | 2.85 | 829.06 | 0.06 | 0.34 | 0.04 | 2.53 | BK-BK/A |

(b)

| Alignment ID | Ionization Mode | Metabolite name | Average RT (min) | Average M/z | BK-0/A TCS | BK-P/A TCS | FDR adjusted  p-value | Log_2_ fold change | Upregulated in |
| --- | --- | --- | --- | --- | --- | --- | --- | --- | --- |
| 21 | Positive | Unknown | 5.38 | 77.04 | 2.11 | 6.88 | 0.04 | 1.70 | BK-P/A |
| 39 | Positive | Unknown | 5.36 | 93.07 | 2.71 | 8.58 | 0.04 | 1.66 | BK-P/A |
| 84 | Positive | Unknown | 8.19 | 120.08 | 0.26 | 0.75 | 0.02 | 1.52 | BK-P/A |
| 90 | Positive | Unknown | 5.37 | 121.06 | 48.91 | 154.74 | 0.03 | 1.66 | BK-P/A |
| 144 | Positive | Unknown | 10.55 | 141.02 | 0.29 | 1.23 | 0.01 | 2.08 | BK-P/A |
| 213 | Positive | Unknown | 8.27 | 160.08 | 0.22 | 0.65 | 0.05 | 1.54 | BK-P/A |
| 302 | Positive | Unknown | 2.17 | 189.12 | 1.34 | 3.17 | 0.03 | 1.25 | BK-P/A |
| 342 | Positive | Unknown | 2.07 | 203.15 | 1.17 | 2.82 | 0.04 | 1.27 | BK-P/A |
| 348 | Positive | Unknown | 8.18 | 206.08 | 0.28 | 1.84 | 0.05 | 2.70 | BK-P/A |
| 216 | Negative | Unknown | 6.96 | 216.09 | 0.30 | 0.74 | 0.03 | 1.33 | BK-P/A |
| 403 | Positive | Unknown | 4.03 | 221.09 | 0.09 | 0.28 | 0.04 | 1.62 | BK-P/A |
| 441 | Positive | Unknown | 7.60 | 231.08 | 0.12 | 0.25 | 0.04 | 1.10 | BK-P/A |
| 298  489 | Negative Positive | Unknown | 10.55 | 241.07  243.09 | 1.40  10.18 | 6.22  59.07 | 0.01  0.02 | 2.15  2.54 | BK-P/A |
| 508 | Positive | Unknown | 10.73 | 249.05 | 0.52 | 1.50 | 0.02 | 1.53 | BK-P/A |
| 526 | Positive | Unknown | 8.19 | 252.09 | 0.73 | 1.96 | 0.05 | 1.42 | BK-P/A |
| 539 | Positive | Unknown | 9.14 | 257.10 | 1.11 | 2.73 | 0.05 | 1.29 | BK-P/A |
| 560 | Positive | Unknown | 11.91 | 261.22 | 3.84 | 8.58 | 0.02 | 1.16 | BK-P/A |
| 577 | Positive | Unknown | 9.21 | 266.08 | 0.04 | 0.10 | 0.03 | 1.52 | BK-P/A |
| 628 | Positive | Unknown | 6.72 | 276.05 | 0.12 | 0.30 | 0.03 | 1.26 | BK-P/A |
| 667 | Positive | Unknown | 11.55 | 285.19 | 1.20 | 2.08 | 0.02 | 0.80 | BK-P/A |
| 669 | Positive | Unknown | 7.58 | 287.08 | 0.15 | 0.67 | 0.03 | 2.20 | BK-P/A |
| 726 | Positive | Unknown | 11.66 | 301.22 | 1.76 | 3.21 | 0.04 | 0.87 | BK-P/A |
| 738 | Positive | Unknown | 2.30 | 305.11 | 0.01 | 0.03 | 0.02 | 1.53 | BK-P/A |
| 502 | Negative | Unknown | 6.73 | 313.13 | 0.03 | 0.25 | 0.04 | 2.85 | BK-P/A |
| 811 | Positive | Unknown | 10.55 | 327.04 | 0.12 | 0.57 | 0.01 | 2.22 | BK-P/A |
| 597 | Negative | Unknown | 8.02 | 351.03 | 0.17 | 0.50 | 0.03 | 1.52 | BK-P/A |
| 893 | Positive | Unknown | 10.96 | 353.25 | 2.13 | 4.04 | 0.04 | 0.93 | BK-P/A |
| 912 | Positive | Unknown | 11.68 | 359.22 | 0.82 | 1.76 | 0.01 | 1.09 | BK-P/A |
| 927 | Positive | Unknown | 11.60 | 363.25 | 0.92 | 1.99 | 0.03 | 1.12 | BK-P/A |
| 934 | Positive | Unknown | 11.22 | 367.27 | 49.51 | 109.67 | 0.03 | 1.15 | BK-P/A |
| 954 | Positive | Unknown | 7.28 | 375.13 | 0.05 | 0.20 | 0.05 | 1.89 | BK-P/A |
| 956 | Positive | Unknown | 11.57 | 375.21 | 1.94 | 3.30 | 0.04 | 0.77 | BK-P/A |
| 971 | Positive | Unknown | 11.19 | 381.25 | 11.32 | 19.91 | 0.04 | 0.81 | BK-P/A |
| 695 | Negative | Unknown | 11.30 | 385.20 | 0.83 | 2.62 | 0.05 | 1.66 | BK-P/A |
| 783 | Negative | Unknown | 10.78 | 425.17 | 0.17 | 0.35 | 0.05 | 1.05 | BK-P/A |
| 878 | Negative | Unknown | 6.75 | 471.08 | 0.11 | 0.20 | 0.04 | 0.93 | BK-P/A |
| 1307 | Positive | Unknown | 7.24 | 495.05 | 0.01 | 0.04 | 0.04 | 1.77 | BK-P/A |
| 934 | Negative | Unknown | 11.08 | 498.21 | 0.08 | 0.17 | 0.04 | 1.07 | BK-P/A |
| 1332 | Positive | Unknown | 10.02 | 506.22 | 0.57 | 1.94 | 0.05 | 1.76 | BK-P/A |
| 1333 | Positive | Unknown | 10.55 | 507.15 | 0.11 | 0.91 | 0.02 | 3.09 | BK-P/A |
| 991 | Negative | Unknown | 9.99 | 533.18 | 0.83 | 2.98 | 0.01 | 1.85 | BK-P/A |
| 999 | Negative | Unknown | 10.83 | 539.24 | 0.04 | 0.20 | 0.03 | 2.18 | BK-P/A |
| 1433 | Positive | Unknown | 9.73 | 564.36 | 5.52 | 11.76 | 0.03 | 1.09 | BK-P/A |
| 1438 | Positive | Unknown | 9.72 | 569.31 | 1.06 | 2.46 | 0.04 | 1.21 | BK-P/A |
| 1706 | Positive | Unknown | 10.84 | 863.40 | 0.02 | 0.04 | 0.05 | 1.38 | BK-P/A |
| 1240 | Negative | Unknown | 11.14 | 1019.50 | 0.00 | 0.01 | 0.04 | 2.90 | BK-P/A |

(c)

| Alignment ID | Ionization Mode | Metabolite name | Average RT (min) | Average M/z | BK-BK/A TCS | BK-P/A TCS | FDR adjusted  p-value | Log_2_ fold change | Upregulated in |
| --- | --- | --- | --- | --- | --- | --- | --- | --- | --- |
| 57 | Positive | Unknown | 6.58 | 103.05 | 180.52 | 91.27 | 0.05 | -0.98 | BK-BK/A |
| 114 | Positive | Unknown | 8.27 | 132.08 | 6.55 | 2.18 | 0.03 | -1.59 | BK-BK/A |
| 156 | Positive | Unknown | 8.27 | 144.08 | 12.00 | 4.40 | 0.01 | -1.45 | BK-BK/A |
| 160 | Positive | Unknown | 8.27 | 146.06 | 43.31 | 15.18 | 0.02 | -1.51 | BK-BK/A |
| 210 | Positive | Unknown | 8.27 | 159.09 | 10.29 | 3.67 | 0.01 | -1.49 | BK-BK/A |
| 213 | Positive | Unknown | 8.27 | 160.08 | 1.53 | 0.65 | 0.03 | -1.24 | BK-BK/A |
| 94 | Negative | Phenylalanine | 6.56 | 164.07 | 10.90 | 3.17 | 0.05 | -1.78 | BK-BK/A |
| 247 | Positive | Unknown | 8.27 | 170.06 | 6.83 | 2.33 | 0.02 | -1.55 | BK-BK/A |
| 341 | Positive | Unknown | 4.02 | 203.08 | 0.66 | 0.27 | 0.01 | -1.27 | BK-BK/A |
| 180  347 | Negative  Positive | Tryptophan | 8.25 | 203.08  205.10 | 71.51  455.86 | 17.55  164.02 | 0.02  0.03 | -2.03  -1.47 | BK-BK/A |
| 403 | Positive | Unknown | 4.03 | 221.09 | 0.77 | 0.28 | 0.04 | -1.45 | BK-BK/A |
| 455 | Positive | Unknown | 7.03 | 235.07 | 0.36 | 0.15 | 0.04 | -1.28 | BK-BK/A |
| 352 | Negative | Unknown | 11.45 | 259.19 | 11.45 | 5.16 | 0.03 | -1.15 | BK-BK/A |
| 397 | Negative | Unknown | 11.57 | 273.21 | 11.24 | 6.13 | 0.04 | -0.88 | BK-BK/A |
| 704 | Positive | Unknown | 7.53 | 295.12 | 9.55 | 4.35 | 0.04 | -1.13 | BK-BK/A |
| 612 | Negative | Unknown | 7.52 | 355.10 | 4.36 | 1.71 | 0.03 | -1.35 | BK-BK/A |
| 661 | Negative | Unknown | 7.33 | 373.12 | 10.30 | 2.60 | 0.05 | -1.99 | BK-BK/A |
| 678 | Negative | Unknown | 6.71 | 379.03 | 0.68 | 0.29 | 0.03 | -1.25 | BK-BK/A |
| 969 | Positive | Unknown | 7.91 | 381.12 | 0.29 | 0.12 | 0.02 | -1.32 | BK-BK/A |
| 1054 | Positive | Unknown | 7.49 | 411.16 | 1.21 | 0.51 | 0.05 | -1.26 | BK-BK/A |
| 1102 | Positive | Unknown | 7.53 | 427.16 | 7.14 | 3.14 | 0.03 | -1.18 | BK-BK/A |
| 855 | Negative | Unknown | 8.20 | 459.15 | 6.75 | 2.61 | 0.01 | -1.37 | BK-BK/A |
| 875 | Negative | Unknown | 7.13 | 469.19 | 15.38 | 8.16 | 0.05 | -0.91 | BK-BK/A |
| 929 | Negative | Unknown | 6.87 | 497.06 | 0.27 | 0.09 | 0.04 | -1.52 | BK-BK/A |
| 955 | Negative | Unknown | 7.22 | 509.22 | 25.34 | 11.09 | 0.04 | -1.19 | BK-BK/A |
| 967 | Negative | Unknown | 6.89 | 515.09 | 5.12 | 1.77 | 0.05 | -1.53 | BK-BK/A |
| 995 | Negative | Unknown | 8.15 | 534.12 | 0.11 | 0.01 | 0.02 | -4.34 | BK-BK/A |
| 1035 | Negative | Unknown | 6.65 | 571.10 | 0.18 | 0.06 | 0.02 | -1.51 | BK-BK/A |
| 1492 | Positive | Unknown | 6.71 | 608.17 | 0.21 | 0.09 | 0.05 | -1.23 | BK-BK/A |

# Table S2: Compounds identified to confidence level 1 according to the Schymanski scale^1^ in buckwheat samples and the retention times and m/z values are listed. While the entire fragmentation pattern of each compound was used for identification, confirmation fragment 1 and 2 were pulled from the fragmentation pattern to show validation of confirmation.

| Alignment ID | Average RT (min) | Average m/z | Compound name | Ionization | Confirmation fragment 1 | Confirmation fragment 2 | Parent fragment |
| --- | --- | --- | --- | --- | --- | --- | --- |
| 29 | 2.62 | 117.02038 | Succinic acid | [M-H]- | 73.03 | 99.01 | 117.02 |
| 53 | 10.59 | 137.02417 | Salicylic acid | [M-H]- | 93.04 | N/A | 137.02 |
| 94 | 6.56 | 164.0713 | Phenylalanine | [M-H]- | 103.05 | 147.04 | 164.07 |
| 98 | 8.01 | 167.03429 | Vanilic acid | [M-H]- | 108.02 | 152.01 | 167.04 |
| 121 | 3.83 | 180.06648 | Tyrosine | [M-H]- | 92.30 | 163.04 | 180.07 |
| 144 | 2.17 | 191.02008 | Citric acid | [M-H]- | 87.01 | 111.01 | 191.02 |
| 180 | 8.25 | 203.08331 | Tryptophan | [M-H]- | 116.05 | 159.09 | 203.08 |
| 440 | 7.21 | 289.07169 | Catechin | [M-H]- | 125.02 | 245.08 | 289.07 |
| 441 | 7.66 | 289.07214 | Epicatechin | [M-H]- | 125.02 | 245.08 | 289.07 |
| 1068 | 10.41 | 609.14563 | Rutin | [M-H]- | 301.03 | 463.07 | 609.15 |
| 234 | 6.58 | 166.08626 | Phenylalanine | [M+H]+ | 103.05 | 120.08 | 166.08 |
| 278 | 3.91 | 182.08127 | Tyrosine | [M+H]+ | 123.04 | 136.08 | N/A |
| 347 | 8.25 | 205.09746 | Tryptophan | [M+H]+ | 146.06 | 188.07 | 205.15 |
| 686 | 7.13 | 291.08649 | Catechin | [M+H]+ | 123.04 | 139.04 | 291.15 |
| 687 | 7.67 | 291.08673 | Epicatechin | [M+H]+ | 123.04 | 139.04 | 291.08 |

# Table S3: Parameters for LC-HRMSMS data acquisition showing the gradient of the liquid chromatography method (a) and the other chromatographic and mass spectrometric conditions (b).

(a)

|  | \| **Time (min)** \| **% A** \| **% B** \| \| --- \| --- \| --- \| \| 0.00 \| 100 \| 0 \| \| 0.50 \| 100 \| 0 \| \| 4.50 \| 95 \| 5 \| \| 5.00 \| 60 \| 40 \| \| 8.00 \| 60 \| 40 \| \| 10.50 \| 0 \| 100 \| \| 12.50 \| 0 \| 100 \| \| 13.50 \| 100 \| 0 \| \| 15.50 \| 100 \| 0 \| \|  \| |
| --- | --- | --- | --- | --- | --- | --- | --- | --- | --- | --- | --- | --- | --- | --- | --- | --- | --- | --- | --- | --- | --- | --- | --- | --- | --- | --- | --- | --- | --- | --- | --- | --- |

(b)

| Chromatographic conditions | |
| --- | --- |
| Stationary phase | Sigma-Aldrich Discovery HS F5  (150 x 2.1 mm, 3 μm particle size) |
| Mobile phase A | 99.9% v/v H2O, 0.1% v/v formic acid |
| Mobile phase B | 99.9% v/v methanol, 0.1% v/v formic acid |
| Flow rate (ml min^-1^) | 0.350 |
| Column oven T (°C) | 50 |
| Injection volume (μl) | 5 |
| Source parameters | |
| Gas Temp (°C) | 300 |
| Gas Flow (l/min) | 8 |
| Nebulizer (psig) | 35 |
| Sheath Gas Temp (l min^-1^) | 300 |
| Sheath Gas Flow (l min^-1^) | 11 |
| VCap | 3500 |
| Nozzle Voltage (V) | 500 |
| Fragmentor (V) | 360 |
| Skimmer 1 | 65 |
| Octopole RF Peak | 750 |
| Scan parameters: Acquisition Mode AutoMS2 | |
| MS Min Range (m/z) | 50 |
| MS Max Range (m/z) | 1700 |
| MS Scan Rate (spectra/sec) | 5.00 |
| Resolution MS1 | 17.5 K @ m/z 322 |
| MS/MS Min Range (m/z) | 50 |
| MS/MS Max Range (m/z) | 1700 |
| MS/MS Scan Rate (spectra/sec) | 7.00 |
| Isolation Width MS/MS | Narrow (~1.3 amu) |
| Collision Energy Table | |
| Mass | Z1 |
| 0 | 5 |
| 300 | 10 |
| 3000 | 100 |
| Precursor Selection | |
| Max Precursors Per Cycle | 5 |
| Threshold (Abs) | 2000 |
| Threshold (Rel)(%) | 0.010 |
| Precursor abundance based scan speed | No |
| Purity Stringency (%) | 100.000 |
| Purity Cutoff (%) | 30.000 |
| Isotope Model | Common |
| Active exclusion enabled | Yes |
| Active exclusion excluded after (spectra) | 3 |
| Active exclusion released after (min) | 0.30 |
| Sort precursors | By abundance only |
| Reference Masses | |
| Negative ionization | **Positive ionization** |
| 119.03632000 m/z | 121.05087300 m/z |
| 966.00072500 m/z | 922.00979800 m/z |

# Table S4: The mock root exudate mixture was made up of 58 chemical analytical standards of compounds known from literature to be exuded by the roots of plants. Species which were found to exudate each compound and the citation for that species is given in the species column. Full reference information for each species citation can be found in the references section of this supplementary file. The company which sourced each standard is also given.

| Compound name | Molecular formula | Exact mass (Da) | Ontology | Species | Source of chemical standard |
| --- | --- | --- | --- | --- | --- |
| Urea | CH4N2O | 60.0324 | Organic molecular entity | *Arabidopsis thaliana*^2^, *Lactuca sativa* L.^3^ | Sigma-Aldrich |
| Glycine | C2H5NO2 | 75.0320 | Amino acid | *Arabidopsis* thaliana^2^,  Triticum *aestivum* L*.*^4^,  *Brachypodium distachyon*^5^ | Sigma-Aldrich |
| Butyric acid | C4H8O2 | 88.0524 | Organic acid | *Triticum aestivum* L*.*^4^, | Sigma-Aldrich |
| β-AIanine | C3H7NO2 | 89.0477 | Amino acid | *Arabidopsis thaliana*^2^,  *Triticum aestivum* L*.*^4^, *Brachypodium distachyon*^5^, *Zea mays*^6^ | Fluka |
| Oxalic acid | C2H2O4 | 89.9953 | Organic acid | *Triticum aestivum* L*.*^4^ | Merck Millipore |
| Valeric acid | C5H10O2 | 102.0681 | Organic acid | *Triticum aestivum* L*.*^4^ | Fluka |
| Y -Amino butyric acid, GABA | C4H9NO2 | 103.0633 | Amino acid | *Arabidopsis thaliana*^2^,  *Triticum aestivum* L*.*^4^, *Brachypodium distachyon*^5^, *Zea mays*^6^ | Sigma-Aldrich |
| Serine | C3H7NO3 | 105.0426 | Amino acid | *Arabidopsis* thaliana^2^,  Triticum *aestivum* L*.*^4^,  *Brachypodium distachyon*^5^ | Sigma-Aldrich |
| Uracil | C4H4N2O2 | 112.0273 | Nucleic Acids | *Arabidopsis thaliana*^2^ | Sigma-Aldrich |
| Proline | C5H9NO2 | 115.0633 | Amino acid | *Triticum aestivum* L*.*^4^ | Sigma-Aldrich |
| Fumaric acid | C4H4O4 | 116.0110 | Organic acid | *Arabidopsis* thaliana^2^,  Triticum *aestivum* L*.*^4^ | Fluka |
| Valine | C5H11NO2 | 117.0790 | Amino acid | *Arabidopsis thaliana*^2^,  *Triticum aestivum* L*.*^4^, *Brachypodium distachyon*^5^ | Merck Millipore |
| Succinic acid | C4H6O4 | 118.0266 | Organic acid | *Arabidopsis* thaliana^2^,  Triticum *aestivum* L*.*^4^ | Fluka |
| Threonine | C4H9NO3 | 119.0582 | Amino acid | *Arabidopsis thaliana*^2^,  *Triticum aestivum* L*.*^4^, *Brachypodium distachyon*^5^, *Zea mays*^6^ | Sigma-Aldrich |
| Benzoic acid | C7H6O2 | 122.0368 | Organic acid, Phenol | *Arabidopsis thaliana*^2^, *Hordeum vulgare*^7^ | Fluka |
| Erythritol | C4H10O4 | 122.0579 | Sugar alcohol | *Arabidopsis thaliana*^2^, *Zea mays*^6^ | Sigma-Aldrich |
| Pyroglutamic acid | C5H7NO3 | 129.0426 | Amino acid | *Arabidopsis thaliana*^2^,  *Sorghum bicolor* L. Moench^8^ | Sigma-Aldrich |
| Isoleucine | C6H13NO2 | 131.0946 | Amino acid | *Arabidopsis thaliana*^2^,  *Triticum aestivum* L*.*^4^, *Brachypodium distachyon*^5^, *Zea mays*^6^ | Sigma-Aldrich |
| Leucine | C6H13NO2 | 131.0946 | Amino acid | *Triticum aestivum* L*.*^4^, *Brachypodium distachyon*^5^, *Zea mays*^6^ | Sigma-Aldrich |
| Asparagine | C4H8N2O3 | 132.0535 | Amino acid | *Arabidopsis thaliana*^2^,  *Triticum aestivum* L*.*^4^, *Brachypodium distachyon*^5^, *Zea mays*^6^ | Sigma-Aldrich |
| Aspartic acid | C4H7NO4 | 133.0375 | Amino acid | *Triticum aestivum* L*.*^4^, *Brachypodium distachyon*^5^ | Merck Millipore |
| Malic acid | C4H6O5 | 134.0215 | Organic acid | *Triticum aestivum* L*.*^4^ | Merck Millipore |
| Threonic acid | C4H8O5 | 136.0372 | Organic acid | *Arabidopsis thaliana*^2^, *Sorghum bicolor* L. Moench^8^ | Sigma-Aldrich |
| Salicylic acid | C7H6O3 | 138.0317 | Organic acid, Phenol | *Medicago sativa*^9^*,  Triticum aestivum, Avena fatua,* many others^10^ | Fluka |
| Glutamine | C5H10N2O3 | 146.0691 | Amino acid | *Triticum aestivum* L*.*^4^, *Brachypodium distachyon*^5^, *Zea mays*^6^ | Sigma-Aldrich |
| Lysine | C6H14N2O2 | 146.1055 | Amino acid | *Triticum aestivum* L*.*^4^, *Brachypodium distachyon*^5^ | Sigma-Aldrich |
| Glutamic acid | C5H9NO4 | 147.0532 | Amino acid | *Triticum aestivum* L*.*^4^,  *Brachypodium distachyon*^5^, *Zea mays*^6^ | Sigma-Aldrich |
| Methionine | C5H11NO2S | 149.0510 | Amino acid | *Triticum aestivum* L*.*^4^, *Brachypodium distachyon*^5^ | Sigma-Aldrich |
| Ribose | C5H10O5 | 150.0528 | Sugar | *Arabidopsis* thaliana^2^,  Triticum *aestivum* L*.*^4^ | Sigma-Aldrich |
| Dopamine | C8H11NO2 | 153.0790 | Phenol | *Brachypodium distachyon*^11^ | Sigma-Aldrich |
| Histidine | C6H9N3O2 | 155.0695 | Amino acid | *Brachypodium distachyon*^5^, *Oryza sativa*^12^ | Fluka |
| Courmaric acid | C9H8O3 | 164.0473 | Organic acid, Phenol | *Medicago sativa*^9^*,*  *Ageratum conyzoides*^13^*,  Eucalyptus spp.*^14^*,  Triticum vulgare*^15^*,  Avena fatua*^16^ | Fluka |
| Phenylalanine | C9H11NO2 | 165.0790 | Amino acid, Phenol | *Triticum aestivum* L*.*^4^, *Brachypodium distachyon*^5^ | Sigma-Aldrich |
| Vanilic acid | C8H8O4 | 168.0423 | Organic acid, Phenol | *Hordeum* vulgare^7^,  *Medicago sativa*^9^*, Eucalyptus spp.*^14^*,  Triticum vulgare*^15^*,  Avena fatua*^16^*, Fagopyrum esculentum*^17^ | Alfa Aesar |
| Shikimic acid | C7H10O5 | 174.0528 | Organic acid | *Arabidopsis thaliana*^2^*, Oryza sativa*^12^ | Sigma-Aldrich |
| Arginine | C6H14N4O2 | 174.1117 | Amino acid | *Brachypodium distachyon*^5^, *Oryza sativa*^12^ | SAFC Pharma |
| Aldohexose (glucose) | C6H12O6 | 180.0634 | Sugar | *Arabidopsis thaliana*^2^*,*  *Triticum aestivum* L*.*^4^, *Zea mays*^6^ | Sigma-Aldrich |
| Tyrosine | C9H11NO3 | 181.0739 | Amino acid, Phenol | *Triticum aestivum* L*.*^4^, *Brachypodium distachyon*^5^, *Zea mays*^6^ | Fluka |
| Isocitric acid | C6H8O7 | 192.0270 | Organic acid | *Oryza sativa*^12^ | Sigma-Aldrich |
| Citric acid | C6H8O7 | 192.0270 | Organic acid | *Triticum aestivum* L*.*^4^,  *Zea mays*^6^ | Sigma-Aldrich |
| Ferulic acid | C10H10O4 | 194.0579 | Organic acid, Phenol | *Hordeum vulgare*^7^,  *Medicago sativa*^9^, *Ageratum conyzoides*^13^*,  Triticum vulgare*^15^*,  Avena fatua*^16^*, Fagopyrum tataricum*^18^ | Fluka |
| Syringic acid | C9H10O5 | 198.0528 | Organic acid, Phenol | *Medicago sativa*^9^*, Eucalyptus spp.*^14^*, Triticum vulgare*^15^*,  Avena fatua*^16^ | Sigma-Aldrich |
| Lauric acid | C12H24O2 | 200.1776 | Fatty acid | *Arabidopsis thaliana*^2^*, Lactuca sativa* L.^3^ | Sigma-Aldrich |
| Tryptophan | C11H12N2O2 | 204.0899 | Amino acid | *Oryza sativa*^12^,  *Fagopyrum esculentum*^19^ | Sigma-Aldrich |
| Jasmonic acid | C12H18O3 | 210.1256 | Organic acid | *Triticum aestivum, Avena fatua,* many others^10^ | Sigma-Aldrich |
| N-acetyl-D-mannosamine | C8H15NO6 | 221.0899 | Sugar | *Arabidopsis thaliana*^2^ | Sigma-Aldrich |
| Cystathionine | C7H14N2O4S | 222.0674 | Amino acid | *Triticum aestivum* L*.*^4^ | Sigma-Aldrich |
| 3,5-Di-tert-butyl-4-hydroxybenzoic acid | C15H22O3 | 250.1569 | Organic acid, Phenol | None - synthetic internal standard | Sigma-Aldrich |
| Palmitic acid | C16H32O2 | 256.2402 | Fatty acid | *Arabidopsis thaliana*^2^*, Fagopyrum esculentum*^20^ | Sigma-Aldrich |
| Stearic acid | C18H36O2 | 284.2715 | Fatty acid | *Arabidopsis thaliana*^2^*, Fagopyrum esculentum*^20^ | Sigma-Aldrich |
| Catechin | C15H14O6 | 290.0790 | Flavonoid, Phenol | *Fagopyrum esculentum*^21^ | Fluka |
| Epicatechin | C15H14O6 | 290.0790 | Flavonoid, Phenol | *Fagopyrum tataricum*^18^ | Sigma-Aldrich |
| Quercetin | C15H10O7 | 302.0427 | Flavonoid, Phenol | *Fagopyrum esculentum*^21^ | Sigma-Aldrich |
| Arachidic acid | C20H40O2 | 312.3028 | Fatty acid | *Fagopyrum esculentum*^20^ | Sigma-Aldrich |
| Myricetin | C15H10O8 | 318.0376 | Flavonoid, Phenol | *Fagopyrum esculentum*^21^ | Sigma-Aldrich |
| Dihexose  (sucrose/maltose) | C12H22O11 | 342.1162 | Sugar | *Arabidopsis thaliana*^2^*, Zea mays*^6^  *Triticum aestivum* L*.*^4^ | Sigma-Aldrich |
| α tocopherol | C29H50O2 | 430.3811 | Lipid, Phenol | *Arabidopsis thaliana*^2^ | Sigma-Aldrich |
| Rutin | C27H30O16 | 610.1534 | Flavonoid, Phenol, Glycoside | *Fagopyrum tataricum*^18^ | Sigma-Aldrich |

# Table S5: MS DIAL pre-processing parameters

| MS-DIAL ver. 4.9.221218 | | |
| --- | --- | --- |
| MS1 Data type | Centroid | |
| MS2 Data type | Centroid | |
| Ion mode | Negative & Positive | |
| Target | Metablomics | |
| Mode | ddMSMS | |
| Data collection parameters | | |
| Retention time begin (min) | 1.8 | |
| Retention time end (min) | 15 | |
| Mass range begin (m/z) | 50 | |
| Mass range end (m/z) | 1700 | |
| MS2 mass range begin (m/z) | 50 | |
| MS2 mass range end (m/z) | 1700 | |
| Centroid parameters | | |
| MS1 tolerance (Da) | 0.01 | |
| MS2 tolerance (Da) | 0.025 | |
| Isotope recognition | | |
| Maximum charged number | 2 | |
| Peak detection parameters | | |
| Smoothing method | Linear Weighted Moving Average | |
| Smoothing level (points) | 6 | |
| Minimum peak width (points) | 6 | |
| Minimum peak height (counts) | 1000 | |
| Peak spotting parameters | | |
| Mass slice width | 0.1 | |
| Deconvolution parameters | | |
| Sigma window value | | 0.5 |
| MS2 Dec amplitude cut off (counts) | | 0 |
| Exclude after precursor | | TRUE |
| Keep isotope until (Da) | | 0.5 |
| Keep original precursor isotopes | | FALSE |
| MSP file and MS/MS identification setting | | |
| Retention time tolerance (min) | | 0.3 |
| Accurate mass tolerance MS1 (Da) | | 0.01 |
| Accurate mass tolerance MS2 (Da) | | 0.05 |
| Identification score cut off (%) | | 80 |
| Using retention time for scoring | | TRUE |
| Using retention time for filtering | | FALSE |
| Adduct ion setting | | |
| Negative ionization | | **Positive ionization** |
| [M-H]- | | [M+H]+ |
| [M-H2O-H]- | | [M+NH4]+ |
| [M+Cl]- | | [M+Na]+ |
| [M+FA-H]- | | [M+K]+ |
| [M-CO2-H]- | | [M+ACN+H]+ |
|  | | [M+H-H2O]+ |
|  | | [M-NH2]+ |
| Alignment parameters setting | | |
| Reference file | | 20220731_Neg_SplitRoot_SingleExtract_BK_QC_R2.abf  20220730_Pos_SplitRoot_SingleExtract_BK_QC_R3.abf |
| Retention time tolerance (min) | | 0.2 |
| MS1 tolerance (Da) | | 0.015 |
| Retention time factor | | 0.5 |
| MS1 factor | | 0.5 |
| Peak count filter (%) | | 0 |
| N detected in at least one group (%) | | 60 |
| Remove feature based on peak height fold-change | | TRUE |
| Sample average (height, counts) /  blank average (height, counts) | | 5 |
| Keep identified and annotated metabolites | | TRUE |
| Keep removable features and assign the tag for checking | | TRUE |
| Gap filling by compulsion | | TRUE |

# References

1. Schymanski, E. L. *et al.* Identifying Small Molecules via High Resolution Mass Spectrometry: Communicating Confidence. *Environ. Sci. Technol.* **48**, 2097–2098 (2014).

2. Chaparro, J. M. *et al.* Root Exudation of Phytochemicals in Arabidopsis Follows Specific Patterns That Are Developmentally Programmed and Correlate with Soil Microbial Functions. *PLOS ONE* **8**, e55731 (2013).

3. Neumann, G. *et al.* Root exudation and root development of lettuce (Lactuca sativa L. cv. Tizian) as affected by different soils. *Front. Microbiol.* **5**, (2014).

4. Rovira, A. D. Plant root exudates. *The Botanical Review* **35**, 35–57 (1969).

5. Kawasaki, A. *et al.* Microbiome and Exudates of the Root and Rhizosphere of Brachypodium distachyon, a Model for Wheat. *PLOS ONE* **11**, e0164533 (2016).

6. Carvalhais, L. C. *et al.* Root exudation of sugars, amino acids, and organic acids by maize as affected by nitrogen, phosphorus, potassium, and iron deficiency. *Z. Pflanzenernähr. Bodenk.* **174**, 3–11 (2011).

7. Shaposhnikov, A. I., Shakhnazarova, V. Yu., Vishnevskaya, N. A., Borodina, E. V. & Strunnikova, O. K. Aromatic Carboxylic Acids in Barley-Root Exudates and Their Influence on the Growth of Fusarium culmorum and Pseudomonas fluorescens. *Appl Biochem Microbiol* **56**, 344–351 (2020).

8. Miller, S. B., Heuberger, A. L., Broeckling, C. D. & Jahn, C. E. Non-Targeted Metabolomics Reveals Sorghum Rhizosphere-Associated Exudates are Influenced by the Belowground Interaction of Substrate and Sorghum Genotype. *International Journal of Molecular Sciences* **20**, 431 (2019).

9. Ghimire, B. K., Ghimire, B., Yu, C. Y. & Chung, I.-M. Allelopathic and Autotoxic Effects of Medicago sativa—Derived Allelochemicals. *Plants* **8**, 233 (2019).

10. Kong, C.-H. *et al.* Plant neighbor detection and allelochemical response are driven by root-secreted signaling chemicals. *Nat Commun* **9**, 3867 (2018).

11. Novak, V. *et al.* *Reproducible Growth of Brachypodium Distachyon in Fabricated Ecosystems (EcoFAB 2.0) Reveals That Nitrogen Form and Starvation Modulate Root Exudation*. http://biorxiv.org/lookup/doi/10.1101/2023.01.18.524647 (2023) doi:10.1101/2023.01.18.524647.

12. Tawaraya, K. *et al.* Metabolite profiling of shoot extracts, root extracts, and root exudates of rice plant under phosphorus deficiency. *Journal of Plant Nutrition* **36**, 1138–1159 (2013).

13. Batish, D. R., Kaur, S., Singh, H. P. & Kohli, R. K. Nature of interference potential of leaf debris of Ageratum conyzoides. *Plant Growth Regul* **57**, 137–144 (2008).

14. Sasikumar, K., Vijayalakshmi, C. & Parthiban, K. T. Allelopathic effects of four eucalyptus species on redgram (Cajanus cajan L.). *Journal of Tropical Agriculture* **39**, 134–138 (2001).

15. Lodhi, M. A. K., Bilal, R. & Malik, K. A. Allelopathy in agroecosystems: Wheat phytotoxicity and its possible roles in crop rotation. *J Chem Ecol* **13**, 1881–1891 (1987).

16. Iannucci, A., Fragasso, M., Platani, C. & Papa, R. Plant growth and phenolic compounds in the rhizosphere soil of wild oat (Avena fatua L.). *Frontiers in Plant Science* **4**, (2013).

17. Kalinova, J., Vrchotova, N. & Triska, J. Exudation of Allelopathic Substances in Buckwheat Fagopyrum esculentum. *Journal of Agricultural and Food Chemistry* **55**, 6453–6459 (2007).

18. Kim, Y. K. *et al.* Production of phenolic compounds in hairy root culture of tartary buckwheat (Fagopyrum tataricum Gaertn). *J. Crop Sci. Biotechnol.* **12**, 53–57 (2009).

19. Gfeller, A., Glauser, G., Etter, C., Signarbieux, C. & Wirth, J. Fagopyrum esculentum Alters Its Root Exudation after Amaranthus retroflexus Recognition and Suppresses Weed Growth. *Front. Plant Sci.* **9**, (2018).

20. Tsuzuki, E., Yamamoto, Y. & Shimizu, T. Fatty Acids in Buckwheat are Growth Inhibitors. *Annals of Botany* **60**, 69–70 (1987).

21. Kalinova, J. & Vrchotova, N. Level of Catechin, Myricetin, Quercetin and Isoquercitrin in Buckwheat ( *Fagopyrum esculentum* Moench), Changes of Their Levels during Vegetation and Their Effect on The Growth of Selected Weeds. *Journal of Agricultural and Food Chemistry* **57**, 2719–2725 (2009).
